# Decoding the hypoxic injury landscape and hypoxic risk model construction in diabetic kidney disease: a multi-omics study

**DOI:** 10.3389/fbinf.2026.1894439

**Published:** 2026-08-05

**Authors:** Li Jiang, Chien Chieh, Haojun Zhang, Tingting Zhao, Xiai Wu

**Affiliations:** 1 Diabetes Department of Integrated Chinese and Western Medicine, China National Center for Integrated Traditional Chinese and Western Medicine, China-Japan Friendship Hospital, Beijing, China; 2 Mental Health Center of Dongcheng District, Beijing, China; 3 Institute of Clinical Medical Sciences, China-Japan Friendship Hospital, Beijing, China

**Keywords:** diabetic kidney disease, hypoxia injury, machine learning, single-cell transcriptome analysis, spatial transcriptomics, United Kingdom biobank

## Abstract

**Objectives:**

To identify the core hypoxic injury pattern of DKD, construct a DKD risk model based on hypoxic injury-related (HIR) score, and explore the potential therapeutic targets of DKD.

**Methods:**

DKD-related microarray-based transcriptomic analyses, single-nucleus RNA sequencing (snRNA-seq) and spatial transcriptomics were retrieved from the Gene Expression Omnibus (GEO) database. Seven HIR gene sets were obtained from various public databases. Core hypoxic genes were identified using different machine-learning algorithm. LASSO and nomogram were applied to construct a HIR risk score for cellular hypoxic damage. The detailed expression of hub gene would be showed in the single cell and kidney region. The prognostic value of the HIR score was externally validated using plasma proteomics from the United Kingdom Biobank.

**Results:**

Five core hypoxic injury pathways in DKD were identified: Hypoxia, Autophagy, Ferroptosis, Endoplasmic Reticulum (ER) Stress, and Apoptosis. The HIR risk score was constructed based on three hub genes: CASP3, DUSP1, and ZFP36. The HIR score demonstrated high diagnostic efficiency for DKD patients. Higher HIR scores were associated with significantly infiltrated immune cells and poorer kidney function. In United Kingdom Biobank validation, the HIR score significantly improved the prediction of kidney outcomes, renal death, and secondary endpoints beyond demographic and metabolic variables (AUC increments 0.04–0.06), and correlated negatively with eGFR and positively with lipoprotein(a). The calculated tissue-level HIR scores also showed a highly significant and robust increase in the renal microenvironment of BTBR ob/ob mice.

**Conclusion:**

These results provided a predictive model for clinical evaluation in patients with DKD and also a new insight into the role of HIR genes in the pathogenesis of DKD.

## Graphical Abstract

**Figure:**
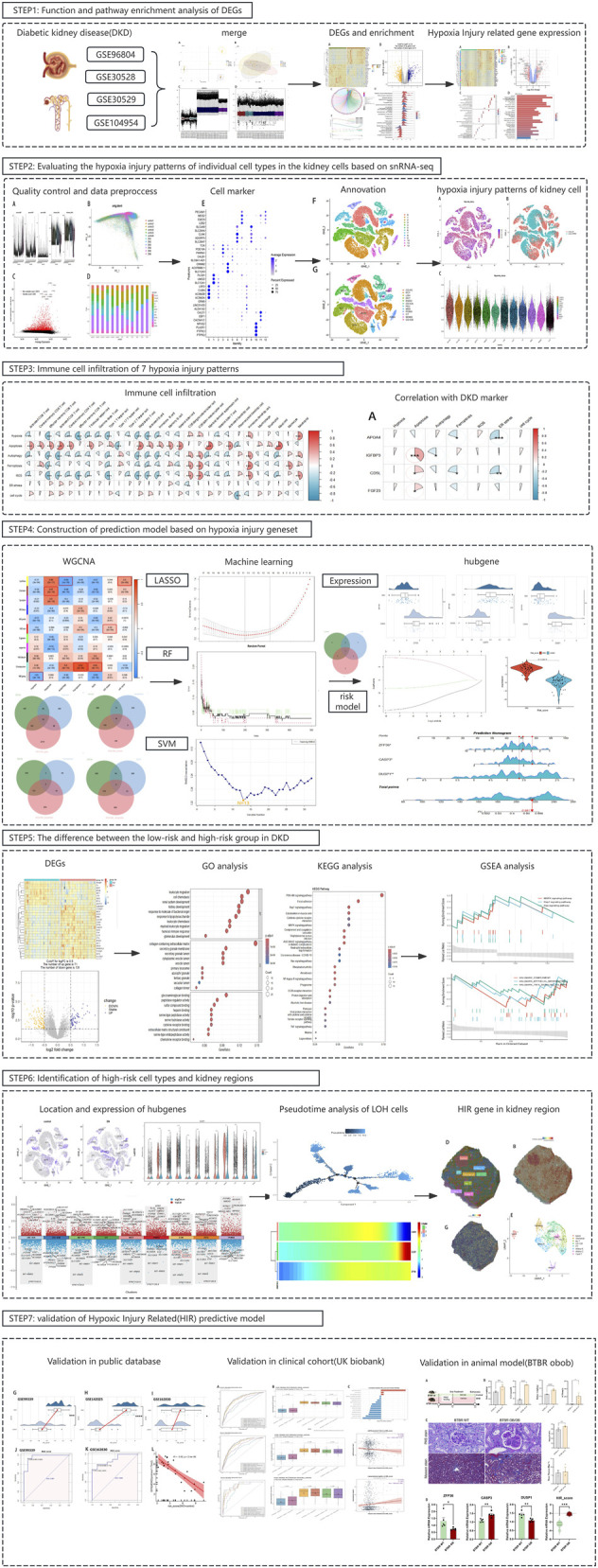

## 1 Intoduction

Diabetic Kidney Disease (DKD) is a renal disease secondary to diabetes, primarily characterized by increased urinary albumin excretion and decreased glomerular filtration rate. It is a common chronic complication of diabetes. Reports indicate that 27%–54% of diabetic patients develop DKD (Afkarian et al., 2016; Liu et al., 2024), leading to substantial healthcare costs. Both the Kidney Disease Outcomes Quality Initiative (KDOQI) of the National Kidney Foundation and the Chinese guidelines for the prevention and treatment of diabetic kidney disease stated that eGFR and/or the urine albumin-to-creatinine ratio (UACR) were the primary clinical diagnostic criteria for DKD (Cai and Chen, 2016; Navaneethan et al., 2024), while kidney biopsy was considered the gold standard for DKD diagnosis. However, eGFR and albuminuria have limitations in sensitivity and specificity, and kidney biopsy’s invasive nature limits its widespread use. The identification of new biomarkers could assist in diagnosing DKD and predicting disease progression.

Chronic hypoxia is an early indicator of renal pathological changes in DKD (Nangaku, 2006). Hyperglycemia induces renal cell hypoxia through various pathological mechanisms. Firstly, hyperglycemia and hyperperfusion lead to glomerular hyperfiltration and increased tubular reabsorption workload, raising oxygen consumption and causing functional hypoxia in the tubules (Singh et al., 2008). Secondly, persistent hyperglycemia triggers oxidative stress, damaging mitochondrial function, disrupting the cellular respiratory chain, and impairing oxygen synthesis (Perco and Mayer, 2018). Thirdly, dysregulation of glucose and lipid metabolism causes endothelial dysfunction, impaired vasodilation, and increased vasoconstriction, leading to increased vascular resistance, impaired blood flow regulation, and reduced renal blood flow and perfusion (A/L B Vasanth Rao et al., 2019). Hypoxic injury plays a crucial role in DKD development, causing decreased intracellular ATP levels, excessive reactive oxygen species production, and activation of apoptotic pathways. This results in the upregulation of fibrotic factors and interstitial fibrosis, further inducing typical glomerular pathological changes such as thickening of the glomerular basement membrane, mesangial cell proliferation, and endothelial cell damage (Shao and Wei, 2025).

Hypoxia-induced biological processes play important roles in DKD progression, including ferroptosis, mitochondrial reactive oxygen species release, mitochondrial autophagy, endoplasmic reticulum-associated stress, apoptosis, and cell cycle arrest (Lee et al., 2020; Zheng et al., 2023). Currently, our understanding of the relationship between hypoxic injury and DKD remains incomplete, including the relative significance of hypoxia-injury pathways involved in DKD progression and their potential synergistic interactions. Given that hypoxia-injury involved multiple cellular pathways and biological factors, bioinformatics methods were essential to identify key molecules and construct disease models. This study aimed to develop a hypoxic injury-related (HIR) risk score—using core HIR molecules involved in DKD progression to predict renal function and immune infiltration in DKD patients. This would reveal the heterogeneity of DKD patients based on hypoxia injury risk characteristics and explore the specific cell types and pathways mediating this heterogeneity.

## 2 Materials and methods

### 2.1 Data acquisition

All datasets were downloaded from the Gene Expression Omnibus (GEO) database (http://www.ncbi.nlm.nih.gov/geo/). We used microarray-based mRNA expression profiles (GSE96804, GSE30528, GSE30529, GSE104954) (Pan et al., 2018; Woronieck et al., 2011; Grayson et al., 2018) for initial analysis, including 63 control samples and 67 DKD samples, all from microdissected human kidney samples covering both glomerular and tubular tissues. The single-nucleus RNA sequencing (snRNA-seq) dataset included 6 control samples and 6 DKD samples, merged from GSE131882 and GSE195460 (Wilson et al., 2019; Muto et al., 2021; Wilson et al., 2022), resulting in 54,112 snRNA-seq. The spatial transcriptomics data originated from GSE261545. Details of the collected datasets can be found in Supplementary Table S1. Key regulatory genes for the 7 hypoxic injury modes were obtained from various sources, including the KEGG database (KEGG.db v4.0), GSEV-Molecular Signatures Database, Genecard database, DisGeNet database, Reactome database, and Comparative Toxicogenomics Database. This included 330 genes related to hypoxia response (hypoxia), 330 genes related to apoptosis (apoptosis), 265 genes related to mitochondrial autophagy (autophagy), 65 genes related to ferroptosis (ferroptosis), 263 genes related to mitochondrial reactive oxygen species release (ROS), 170 genes related to endoplasmic reticulum stress (ER-stress), and 157 genes related to cell cycle arrest (cell cycle). Clinical data for DKD patients were obtained from the Nephroseq v5 online database.

### 2.2 Data processing

We combined the microarray data of mRNA expression profiles using the R packages “limma” and “sva”, and removed batch effects using the ComBat function (Swindell et al., 2019). The distribution patterns of the samples were displayed through box plots, principal component analysis (PCA) plots, and 3D projection plots, as detailed in Supplementary Material 1. Genes with log2FC > 0.5 and P value <0.05 were considered differentially expressed genes (DEGs) (Wang et al., 2023), and volcano plots and heatmaps were generated.

The snRNA-seq and spatial transcriptome data were processed using the “Seurat” package (Xu et al., 2021). High-variable genes expressed in at least 3 cells and marker genes expressed in at least 50 features were extracted. Cells with gene expression <200 or >5000, mitochondrial gene expression >20%, ribosomal gene expression >20%, or hemoglobin gene expression >5% were excluded. The SCTransform function and PCA were used to normalize and scale the raw counts. Unsupervised clustering analysis and uniform manifold approximation and projection (UMAP) were employed to identify discrete cell clusters in the snRNA-seq dataset. Cell annotation information for snRNA was obtained from the K.I.T database (http://humphreyslab.com/SingleCell/) and Cellmarker 2.0 database (Hu et al., 2023), with all clusters and subclusters manually annotated. The annotation for spatial pathological section was referred to the original article (Isnard et al., 2024).

### 2.3 Functional enrichment analysis and pathway activity calculation

We used the R package “clusterProfiler” for KEGG and Gene Ontology (GO) enrichment analysis (Cao et al., 2019). To identify potential biological pathways, a significance threshold of 0.05 was set, and gene set enrichment analysis (GSEA) was conducted using the “GSEABase” package (Yin et al., 2022). The GSVA package was used to explore potential pathway-level changes in gene expression to determine pathway activity differences between samples.

### 2.4 Machine learning and construction of receiver operating characteristic curves

Based on pathway enrichment scores obtained from GSVA, we optimized the selection of gene sets for HIR modules using the weighted gene co-expression network analysis (WGCNA) algorithm (Langfelder and Horvath, 2008). The top 25% of genes by variance were used as clustering templates. We evaluated the predictive ability of seven hypoxic injury modes using the “pROC” package to calculate AUC values (Robin et al., 2011). Three machine learning algorithms were used to screen core hypoxic injury genes: Least Absolute Shrinkage and Selection Operator (LASSO) (D'Angelo et al., 2009), the Support Vector Machine-Recursive Feature Elimination (SVM-RFE) (Xia et al., 2020), and the Regularized Random Forest (RRF) (Pfeifer et al., 2022). The intersection of the results was used for predictive model construction, with coefficients generated by LASSO. The discriminatory ability of the HIR score for distinguishing DKD patients from controls was evaluated using ROC curve analysis with the pROC R package, and model robustness was rigorously assessed through multiple complementary validation strategies: bootstrap resampling (1,000 iterations) for 95% confidence interval estimation, nested cross-validation (5-fold outer, 3-fold inner, repeated 50 times), 10-fold cross-validation (repeated 100 times), leave-one-out cross-validation (LOOCV), Lasso and Ridge regularized logistic regression using the glmnet R package, and permutation testing (1,000 random permutations of group labels), with the overfitting gap defined as the difference between the apparent AUC and the nested cross-validation AUC. A nomogram was drawn to evaluate the model’s predictive ability (Wu et al., 2020).

### 2.5 Immune infiltration analysis

To decipher the cell-type-specific immune landscape within the heterogeneous renal parenchyma, single-sample Gene Set Enrichment Analysis (ssGSEA) was implemented using the GSVA R package framework. The baseline immunogenomic marker sets deployed for calculating the relative infiltration scores were retrieved from the authoritative multi-tissue immune atlas validated by Charoentong et al. (Charoentong et al., 2017; Becht et al., 2016). Crucially, to justify the organ-specific suitability of these signatures for the rigid, fibrotic microenvironment of diabetic kidney disease (DKD), the marker matrices were cross-referenced and benchmarked against established human kidney single-cell and single-nucleus transcriptomic datasets. This tissue-specific alignment successfully accounted for resident myeloid lineages and interstitial stroma-associated immunomodulatory cells while filtering out blood-borne cellular biases. Hierarchical clustering of the infiltration profiles was subsequently executed using the pheatmap architecture via the Ward. D2 method paired with squared Euclidean distance metrics. Statistical variations in differentially infiltrated immune cell lineages between groups were analyzed and visualized using standard exploratory data analysis pipelines.

### 2.6 Single-cell downstream analysis

The “FeaturePlot” function was used to display gene expression locations and quantitative differences. The cell pseudotime analysis was conducted using the BiocGenerics and monocle packages to explore differentiation status and dynamic gene expression. Cell communication analysis was performed using the “cellchat” package to reveal pathway connections between primary cells and other cells (Fang et al., 2022).

### 2.7 Spatial transcriptome analysis

The conditional autoregressive-based deconvolution (CARD) was applied for spatial division and inter-regional difference analysis (Ma and Zhou, 2022). The hub genes would be mapped to specific locations in pathological sections to reflect its actual expression. Regional differential genes would be used for KEGG enrichment analysis to verify the importance of hypoxia response.

### 2.8 HIR score for the prognosis of DKD based on United Kingdom biobank proteomics

We externally validated three hypoxia-related genes (CASP3, DUSP1, ZFP36) using the United Kingdom Biobank, a prospective population-based cohort of approximately 500,000 adults aged 39–70 years recruited between 2006 and 2010, with linked national mortality, hospitalisation and cancer registries. High-throughput plasma proteomics were measured using the Olink Explore 1536 panel in a probability subsample. For our specific implementation, proteins demonstrating missingness rates >20% were excluded from downstream analysis. The circulating levels of the available target proteins matching our core risk components (e.g., CASP3) were extracted to compute the integrated clinical HIR score. Longitudinal hard endpoints—including functional kidney decline, renal death, and secondary macrovascular outcomes—were cross-referenced using ICD-10 registry codes linked to electronic health records to benchmark the fully adjusted incremental predictive metrics. Diabetic kidney disease (DKD) cases were identified through an integrative approach combining ICD-10 codes (E10.2, E11.2, etc.), established clinical guidelines and laboratory criteria (eGFR <60 mL/min/1.73 m^2^ and/or urine albumin-to-creatinine ratio ≥30 mg/g sustained for ≥3 months). After excluding participants with pre-existing kidney failure, missing proteomic data or failing quality control, 918 DKD patients were included. The composite kidney outcome comprised renal death (primary outcome), end-stage kidney disease, sustained eGFR decline ≥40% from baseline, and sustained UACR increase ≥30%. Receiver operating characteristic (ROC) curves assessed predictive performance of individual protein markers alone and in combination with demographic factors (age, sex, etc.) and metabolic traits (HbA1c, glucose, lipids, blood pressure, renal markers). DeLong tests compared AUC differences across models, and 2000-iteration bootstrap analyses estimated AUC confidence intervals. Correlative mapping between the protein abundance encoded by the HIR gene and routine clinical indices employed Spearman rank correlation, with coefficients (β) reported and significance defined as P < 0.05.

### 2.9 The validation of HIR score in animal model

To experimentally validate the calculated hypoxia injury risk (HIR) signature, 8-week-old male black and tan brachyury (BTBR) ob/ob diabetic mice (n = 5) and age-matched male BTBR wild-type (WT) mice (n = 5) were maintained under standard 12-h light/dark cycles with *ad libitum* food and water until ethical euthanasia at 24 weeks of age. Harvested renal tissues were fixed in 4% paraformaldehyde, embedded in paraffin, sectioned at 4 μm and subjected to Periodic Acid-Schiff (PAS) staining to track structural lesions. Concurrently, total RNA was isolated from cryopreserved, mechanically pulverized kidney samples using TRIzol reagent, purified, and reverse-transcribed into cDNA. Quantitative real-time PCR (qRT-PCR) amplification was subsequently executed using SYBR Green Master Mix to evaluate the transcription profiles of the core target genes (ZFP36, DUSP1, and CASP3), with target fold-changes calculated using the comparative 2^−ΔΔCT^ method relative to the internal house-keeping control GAPDH.

## 3 Results

### 3.1 Data merging

The four DKD datasets were merged and normalized after batch effects were removed using the ComBat algorithm. The distribution patterns between normal and DKD samples were visualized using principal component analysis (PCA), box plots, and 3D projection plots (Supplementary Figure S1). These results confirmed that batch effects were successfully removed.

### 3.2 Identification and enrichment analysis of DEGs between control and DKD groups

**FIGURE 1:**
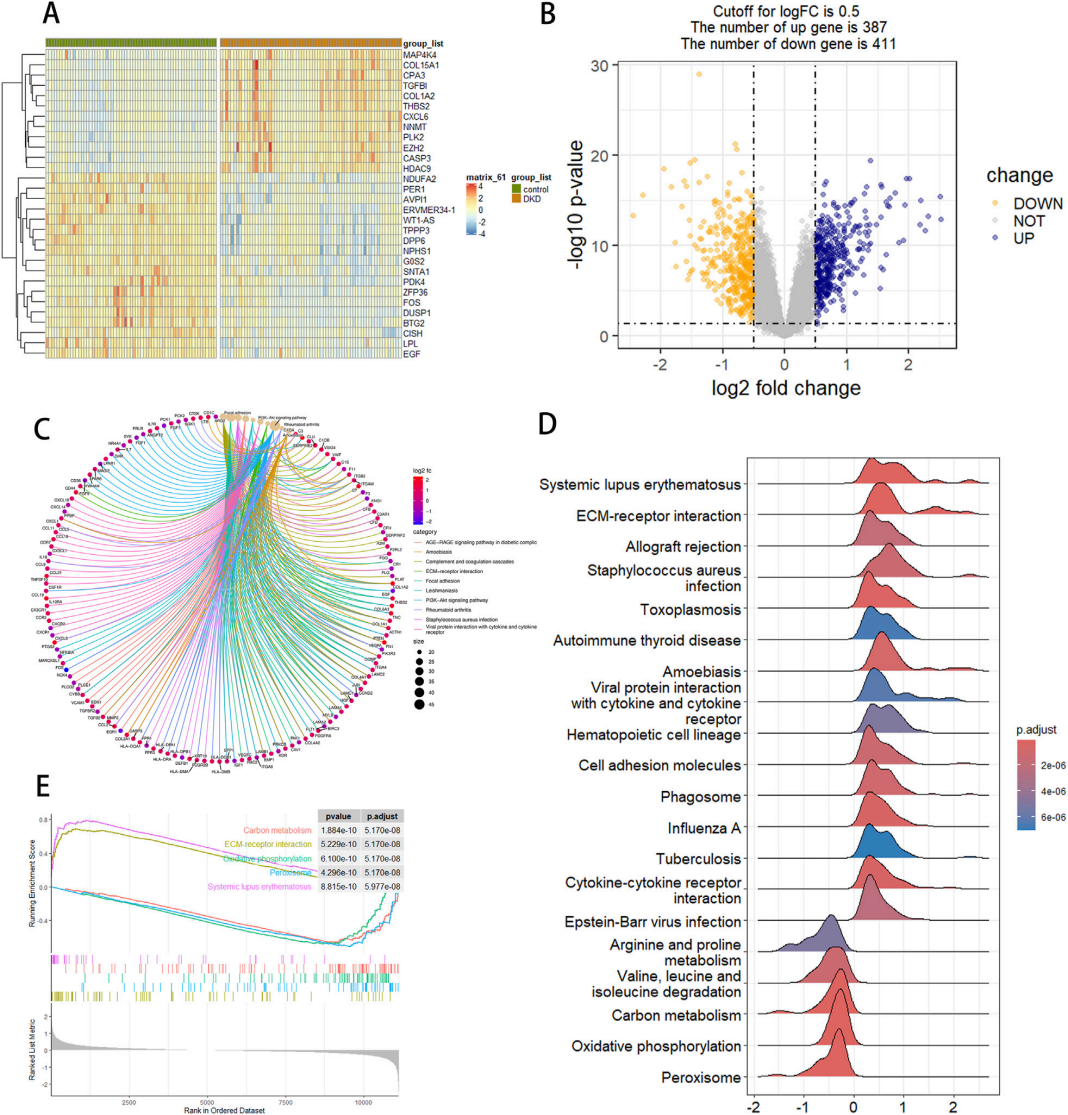
Functional and Pathway Enrichment Analysis of DEGs between Control and DKD Groups **(A)** displays the expression patterns of robust differentially expressed genes (DEGs), and the volcano plot **(B)** highlights significantly upregulated and downregulated transcripts. **(C)** KEGG pathway enrichment analysis revealing that the identified DEGs are predominantly enriched in classical diabetic microvascular injury pathways, including advanced glycation end-products (AGE-RAGE) signaling, ECM-receptor interaction, and MAPK signaling. **(D,E)** Gene Set Enrichment Analysis (GSEA) of MSigDB hallmark gene sets, showing prominent activation of the Inflammatory Response, Complement, and Hypoxia cascades in the DKD group. Note: Bulk RNA-seq data were merged from four independent cohorts (GSE96804, GSE30528, GSE30529, GSE104954). DEGs were defined by log_2FC > 0.5and adjusted P < 0.05 using the limma package with ComBat batch correction.

The significant differences were observed between normal and DKD samples, identifying 798 DEGs. The volcano and heatmaps (Figures 1A,B) indicated that 387 genes were upregulated and 411 genes were downregulated in the DKD group. The pathway enriched were most associated with metabolism such as “Tryptophan metabolism,” “Fatty acid degradation,” and “Tyrosine metabolism” (Figure 1C). GSEA has revealed the potential pathways such as “ECM-receptor interaction” and “Cell adhesion molecules” (Figure 1D). The top activated pathway was “ECM-receptor interaction,” while the top three suppressed pathways were “Carbon metabolism,” “Oxidative phosphorylation,” and “Peroxisome” (Figure 1E).

### 3.3 Identification and enrichment analysis of HIR DEGs in control and DKD groups

**FIGURE 2:**
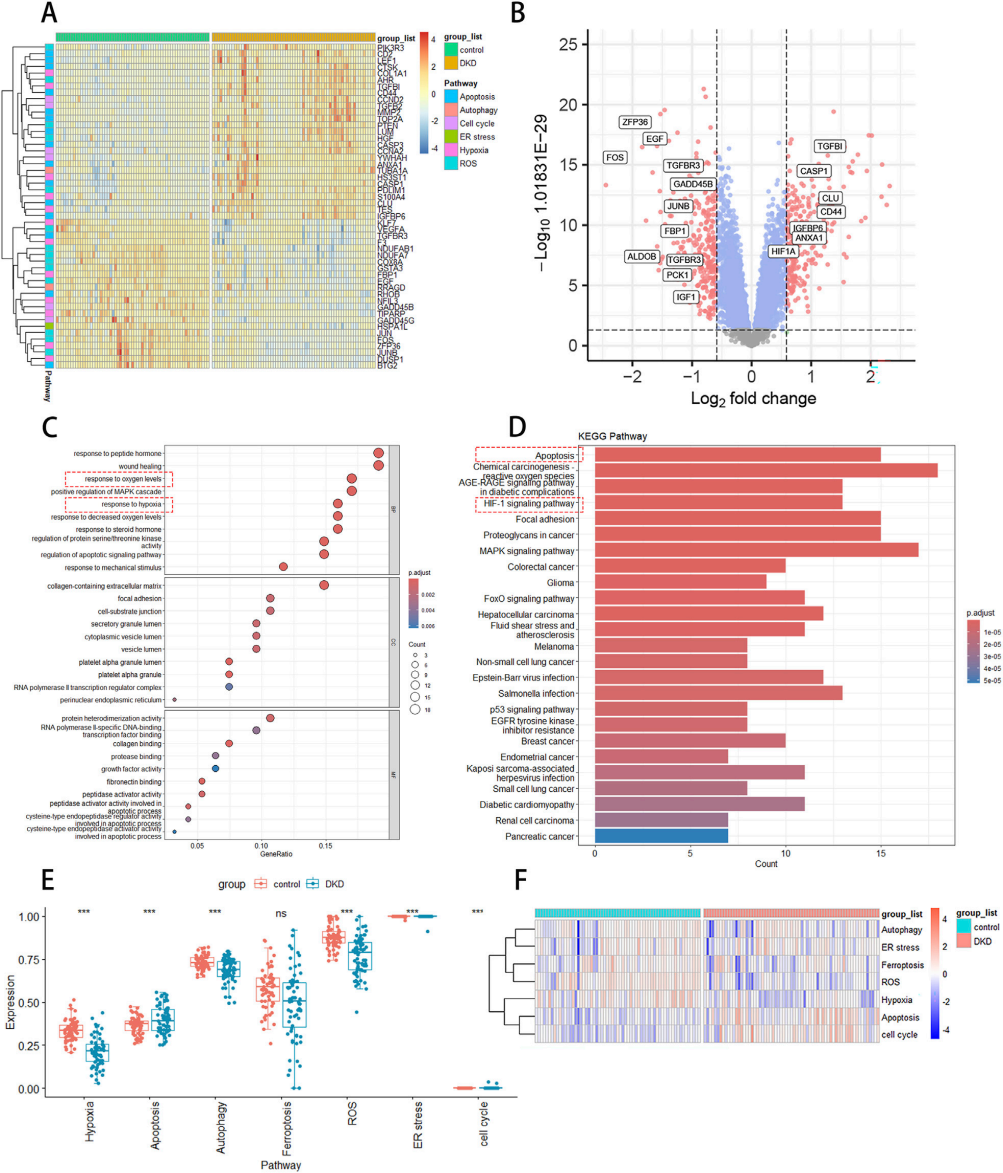
Characterization and Activation Profiles of Hypoxic Injury-Related (HIR) Signatures in Dissected Kidney Tissues. **(A,B)** Differential expression profiling focused on hypoxic injury-related (HIR) genes. Supervised hierarchical clustering **(A)** and volcano plot **(B)** isolate the core hypoxic elements driving the phenotypic transition from healthy controls to DKD. **(C,D)** Functional annotation of HIR DEGs via GO **(C)** and KEGG **(D)** enrichment analyses, revealing substantial involvement in response to oxygen levels, cellular response to hypoxia, and apoptosis regulation. **(E,F)** Pathway activity score mapping. GSEA **(E)** illustrates the significant enrichment of cellular hypoxia hallmarks, while Gene Set Variation Analysis (GSVA) **(F)** quantifies the distinct activation matrix of 7 predefined HIR pathways across individual patient samples.

We then focused the differences in HIR genes between normal controls and DKD patients. The heatmap and volcano plot (Figures 2A,B) showed the key HIR genes which expressed deferentially in DKD patients. GO enrichment indicated that HIR DEGs were enriched in biological processes associated with hypoxia regulation (Figure 2C). KEGG analysis revealed that DEGs participated in downstream regulation of hypoxia like “Apoptosis” and the “HIF-1 signaling pathway” (Figure 2D). GSVA showed that 2 pathways, apoptosis and cell cycle, were upregulated in DKD patients, while 4 HIR pathways showed decreased activity in DKD (hypoxia, autophagy, ROS, ER stress) (Figure 2E). Furthermore, a heatmap of pathway activation scores clearly demonstrated distinct clusters between the two groups (Figure 2F). In agreement with the GSVA results, pathways including hypoxia, autophagy, ROS, and ER stress exhibited consistently reduced activity patterns in the DKD group compared to normal controls, whereas apoptosis and cell cycle pathways were visibly upregulated in DKD samples.

After data pre-processing (Supplementary Figure S2), 12 cell types were manually identified: mesenchymal cells (MES), glomerular parietal epithelial cells (PEC), proximal convoluted tubular cells (PCT), Loop of Henle cells (LOH), distal convoluted tubular cells (DCT), convoluted tubular cells (CT), collecting duct-principal cells (CD-PC), collecting duct-intercalated cell type A (CD-ICA), collecting duct-intercalated cell type B (CD-ICB), podocytes (PODO), endothelial cells (ENDO), and monocytes (MONO). The AUCell analysis showed the activity of HIR geneset decreased in the following order: CD-ICA, CD-ICB, PEC, CD-PC, ENDO, PODO, LOH, DCT, CT, MES, MONO, PCT (Supplementary Figure S3).

The proportions of several immune cells were found to be increased in DKD patients in Supplementary Figure S4. The Hypoxia, Autophagy, Ferroptosis, and ROS geneset, had a significant negative correlation with the infiltration of many immune cells like effector memory CD8 T cells, activated CD4 T cells and activated dendritic cells, while the Apoptosis geneset showed a positive correlation. The biomarkers APOA4, IGFBP3, CD5L, and FGF23 were independent risk factors for composite endpoints of DKD microalbuminuria, doubling of serum creatinine, ESRD, and cardiovascular events (Titan et al., 2011; AgarwalR et al., 2014). Supplementary Figure S5 showed that Apoptosis was significantly positively correlated with three biomarkers (IGFBP3, CD5L, FGF23).

### 3.4 Analysis of the module closely related to HIR geneset in DKD

The WGCNA analysis revealed that the yellow module exhibited the strongest correlation with Autophagy and cell cycle, while the brown module showed the strongest correlation with Apoptosis, the red module with ER stress, the turquoise module with ROS and Ferroptosis, and the grey module with Hypoxia (Supplementary Figure S6). The corresponding intersections were depicted in Supplementary Figure S7. Summarizing the intersections across the seven group genes, KEGG analysis showed significant expression of “AGE-RAGE signaling pathway in diabetic complications”, “Apoptosis”, and “reactive oxygen species”, consistent with the enrichment results of HIR geneset.

In total of 32 genes were subjected to LASSO regression, resulting in the identification of 11 genes, namely, DUSP1, NFIL3, PIK3R3, S100A4,ATF3, IGF1, CASP3, CLU, IGFBP6, ZFP36, HIF1A as hub genes in the DKD dataset (Supplementary Figures S8A–C). By SVM-RFE analysis, 13 genes have been calculated as the best model (Supplementary Figures S8D,E) and ranked by importance. By RRF analysis, 5 genes has been filtered out (Supplementary Figures S8G,H) and ranked by accuracy.

**FIGURE 3:**
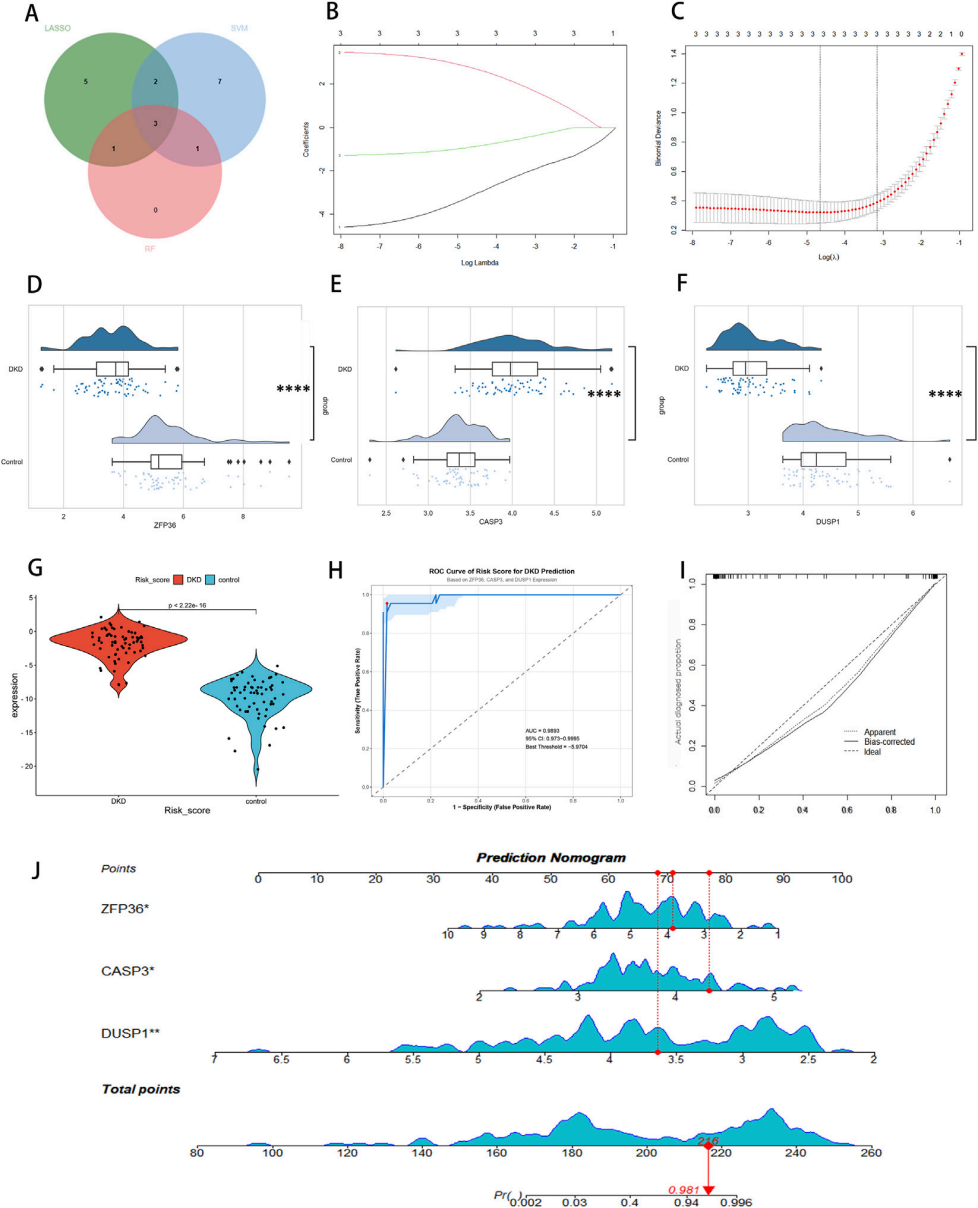
Construction and Evaluation of an Optimization-Driven 3-Gene HIR Diagnostic Risk Model and Diagnostic Nomogram. **(A)** The intersection gene of three machine learning method. **(B)** Distribution of LASSO coefficients for differential genes. **(C)** Ten-fold cross-validation for tuning parameter selection in the LASSO model. **(D–F)** The raincloud plots showing the expression of four 3 HIR genes in control and DKD patients (ZFP36, CASP3, DUSP1). **(G)** The violin plot displaying the HIR risk score distribution between control and DKD patients. **(H)** ROC curve analysis of the 3 HIR genes in DKD. **(I)** Calibration curve of Nomogram-predicted probability. **(J)** Nomogram of the 3 HIR genes in DKD: sample GSM756994 was utilized as a representative instance to demonstrate the clinical application of the predictive nomogram. ****P < 0.0001.

Using the intersection of three machine learning algorithms, we identified three core HIR genes: ZFP36, CASP3, and DUSP1 (Figure 3A). LASSO regression analysis determined the factors for constructing the HIR risk score. The formula was established as follows: ZFP36 expression * (−0.90653) + CASP3 expression * (2.75490) + DUSP1 expression * (−3.16704) (Figures 3B,C). Compared to normal controls, ZFP36 and DUSP1 were significantly downregulated, while CASP3 was upregulated in the kidney tissues of DKD patients (Figures 3D–F). The HIR score was significantly increased in the DKD group compared to the control group (Figure 3G). The higher the HIR score was, the higher the prediction efficiency for DKD would be. In the test set, the average HIR score of the control group was −9.80, with a median score of −9.10 and a standard deviation of 2.94. The average HIR score of the DKD group was −1.84, with a median score of −1.49 and a standard deviation of 2.16. The ROC curve demonstrated that the HIR score exhibited good discriminatory performance for diagnosing DKD, with an apparent AUC of 0.9893 (95% bootstrap CI: 0.9730–0.9995). At the optimal threshold of −5.9704 (determined by Youden index), the model achieved a sensitivity of 95.52% and specificity of 98.41% (Figure 3H). Comprehensive cross-validation confirmed model robustness, with nested cross-validation AUC of 0.9897, 10-fold cross-validation AUC of 0.9892, and leave-one-out cross-validation AUC of 0.9893, yielding a negligible overfitting gap of −0.0004 (Supplementary Figure S9). The Nomogram-based model also demonstrated high weight and predictive capability for the three hub genes (Figures 3I,J).

### 3.5 Identification of DEGs grouped by HIR risk score

According to the median HIR risk score, DKD patients were divided into two groups. We identified 202 DEGs between the low-risk and high-risk groups, including 71 upregulated and 131 downregulated genes (Supplementary Figures S10A,B). GO analysis revealed that the DEGs were enriched in typical process of kidney disease formation like “renal system development” “leukocyte migration” and “glycosaminoglycan binding” (Supplementary Figure S10C). KEGG analysis indicated some inflammatory process like “AGE-RAGE” and “PI3K-Akt signaling pathway” (Supplementary Figure S10D). The GSEA results showed that the MAPK and Ras signaling pathways were activated (Supplementary Figures S10E,F).

The high-risk group exhibited higher levels of immune cell infiltration compared to the low-risk group (Supplementary Figure S11A). Additionally, we found significant positive correlations between the HIR risk score and three core hypoxia genes with certain immune cell types, particularly Type 17 helper T cells and plasmacytoid dendritic cells (Supplementary Figure S11B).

### 3.6 Validation of HIR risk score in public database

**FIGURE 4:**
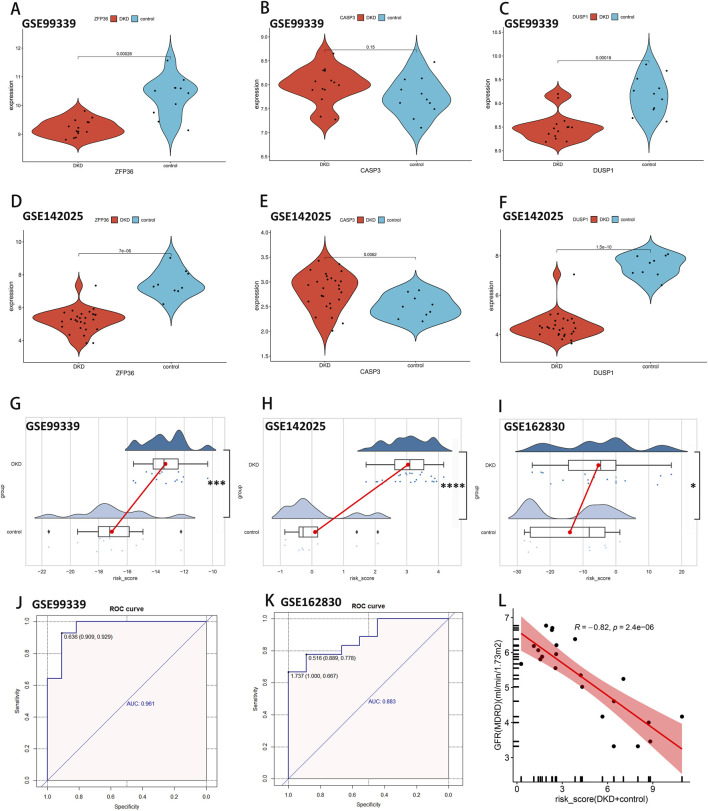
Robust External Validation of Hub Genes and HIR Risk Scores Across Multiple Microdissected Tissue Cohorts. **(A–F)** Verification of individual gene expression. CASP3, DUSP1, and ZFP36 expression levels were robustly recapitulated in independent external validation cohorts GSE99339 **(A–C)** and GSE142025 **(D–F)**, maintaining strict directional consistency with the training dataset. **(G–I)** Cross-cohort evaluation of the HIR risk score, confirming consistent and significant upregulation of the cumulative score in the DKD group across three separate validation matrices. **(J,K)** ROC curve analyses verifying the outstanding and reproducible diagnostic accuracy (AUC) of the HIR risk model in the respective validation cohorts. **(L)** Scatter plots with Pearson’s correlation analysis illustrating a strong, statistically significant negative association between the HIR risk score and measured Glomerular Filtration Rate (GFR) in patients.

The GSE99339, GSE162830 and GSE142025 were selected as the validation cohort. The expression of three hub core genes (CASP3, ZFP36, DUSP1) showed significant differences in the DKD group (Figures 4A–F) in two validation dataset. The HIR risk score of the DKD group was significantly higher than that of the control group (Figures 4G–I). We generated an ROC curve to evaluate the diagnostic performance of the HIR risk score for DKD, with an AUC of 0.961 and 0.883 (Figures 4J,K). The clinical data showed a significant negative correlation between the risk score and GFR levels (Figure 4L).

### 3.7 Exploration of hub gene expression at single-cell level and spatial transcriptome

**FIGURE 5:**
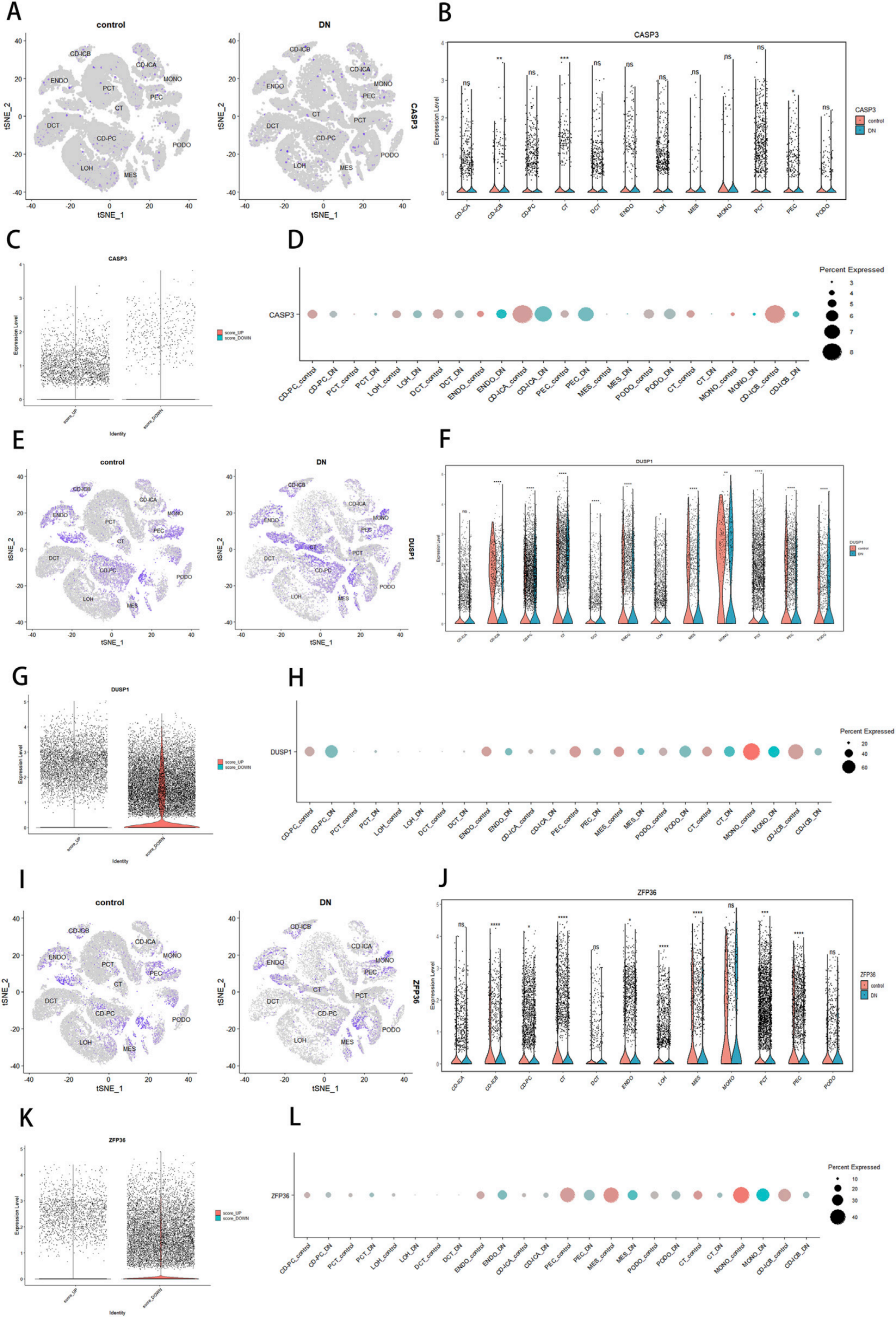
Single-Cell Resolution Atlas Mapping the Specific Microenvironmental Localization of Hub Genes. **(A–D)** Single-cell topography and stratification of CASP3. The UMAP plot **(A)** displays the global distribution across 9 major intrarenal cell types. Split violin plots **(B)** show cell-type-specific differential upregulation in Collecting Duct-Intercalated Cells (CD-ICB), Connective Tissue (CT), and Parietal Epithelial Cells (PEC) in DKD. Quantified bar charts illustrate CASP3 elevation in high HIR score cell types **(C)**, with peak enrichment found in CD-ICA cells **(D)**. **(E–H)** Distribution and modulation matrix of DUSP1. UMAP visualization **(E)** shows concentration in CD-Principal Cells (CD-PC), CT, and Endothelial cells (ENDO). **(F)** highlights significant downregulation in ENDO and Loop of Henle (LOH) cells during DKD. **(G,H)** show downregulated tendencies in high HIR score cell components and minimum enrichment in LOH cells. **(I–L)** Distribution and downregulation topology of ZFP36. **(I)** shows widespread dispersion, while **(J)** confirms robust downregulation across multiple compartments (CD-ICB, CD-PC, CT, ENDO, LOH) under diabetic conditions. **(K,L)** validate its inverse correlation with high HIR scores and its lowest expression tier in LOH cells.

The expression characteristics of 3 hub genes were shown in a single-cell level. CASP3 was found to be dispersed among kidney cells (Figure 5A) and differentially upregulated in CD-ICB, CT, and PEC cells in the DKD group compared to the control group (Figure 5B). It expressed more in high HIR score cell types (Figure 5C) and was most enriched in CD-ICA cells (Figure 5D). DUSP1 was concentrated in CD-PC, CT, and ENDO cells (Figure 5E), but differentially downregulated in ENDO and LOH cells (Figure 5F). It also expressed low in high HIR score cell types (Figure 5G) and was least enriched in LOH cells (Figure 5H). ZFP36 was dispersed among kidney cells (Figure 5I) and differentially downregulated in CD-ICB, CD-PC, CT, ENDO, and LOH cells (Figure 5J). It showed low in HIR score cell types (Figure 5K) and was least enriched in LOH cells (Figure 5L).

**FIGURE 6:**
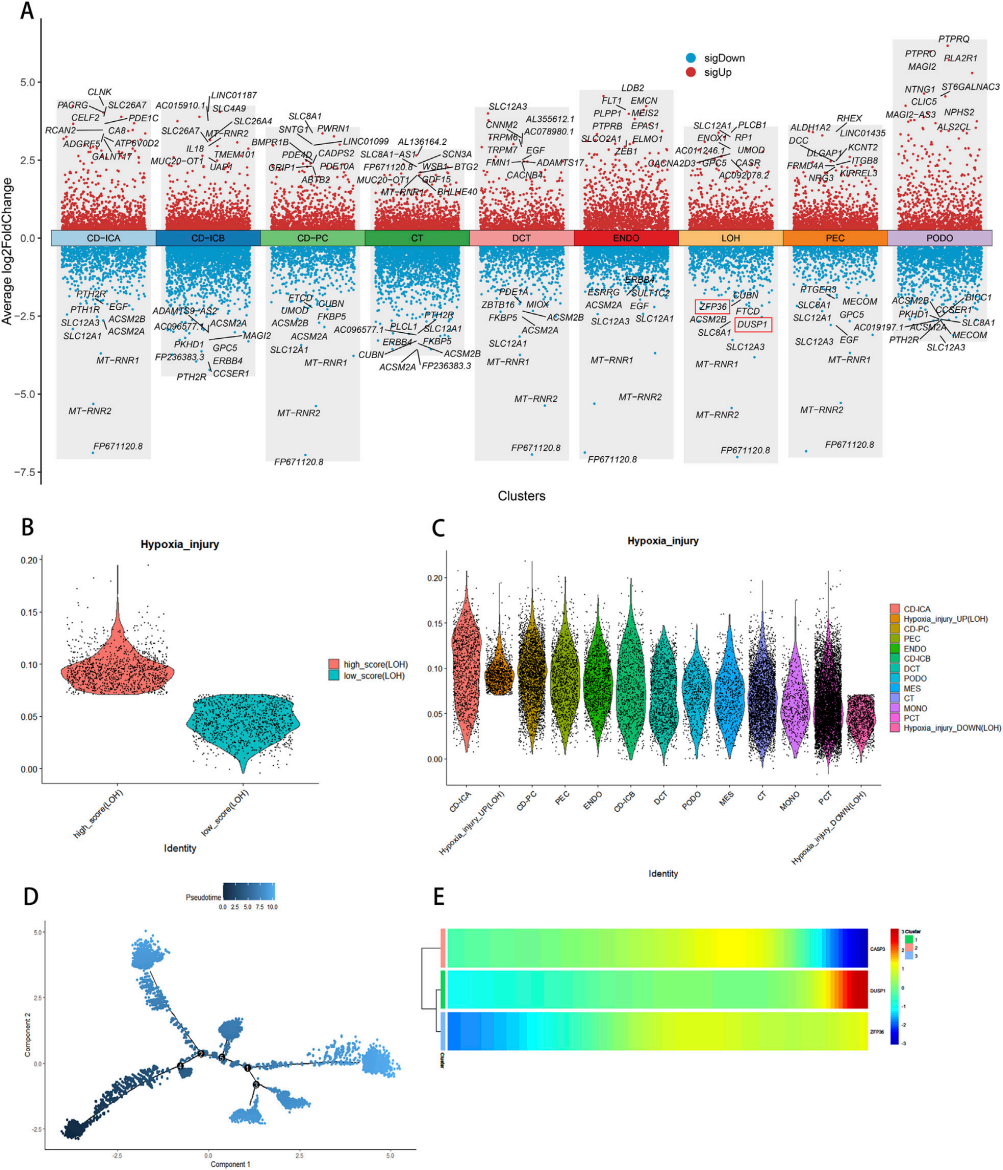
Cellular Trajectory and Pseudotime Kinetics Revealing Hypoxic Injury Susceptibility in the Loop of Henle (LOH). **(A)** Comparative analysis bar chart highlighting that among the 9 defined renal cell types, LOH cells display the most pronounced differential vulnerability and score shift under hypoxic stress. **(B)** High-resolution sub-clustering UMAP of LOH cells, segregating the population into distinct functional or anatomical sub-states. **(C)** Density and gradient ranking of individual LOH cells arranged according to their computed single-cell HIR risk scores, highlighting the progression of cellular injury. **(D)** Monocle2 pseudotime trajectory tree of LOH cells capturing the continuous state transition from healthy/early states to the advanced, diseased phenotypes. **(E)** Kinetic expression curves displaying the continuous, pseudotime-dependent transcriptional enrichment or exhaustion profiles of the 3 hub genes along the identified injury trajectory.

Differential analysis showed that both DUSP1 and ZFP36 were significantly downregulated in LOH cells (Figure 6A). So the LOH cells were segregated into high-risk and low-risk groups based on the median HIR risk score (Figure 6B). The Comparison based on HIR risk scores revealed that the high-risk subgroup of LOH cells ranked by the front (Figure 6C). The pseudotime analysis of LOH unveiled a developmental trajectory progressing from shallow to deep through four pivotal branching points (Figure 6D). The expression of DUSP1 and ZFP36 peaked in mature LOH cells and was lowest in intermediate PCT cells, indicating an enrichment pattern associated with cell differentiation (Figure 6E).

The cell communication revealed that the high-HIR risk subgroup of LOH cells communicated with other cell types through a complex network (Supplementary Figure S12A). Among them, EGF served as a key intermediate pathway for both incoming signaling patterns (Supplementary Figure S12B) and outgoing signaling patterns (Supplementary Figure S12C).

**FIGURE 7:**
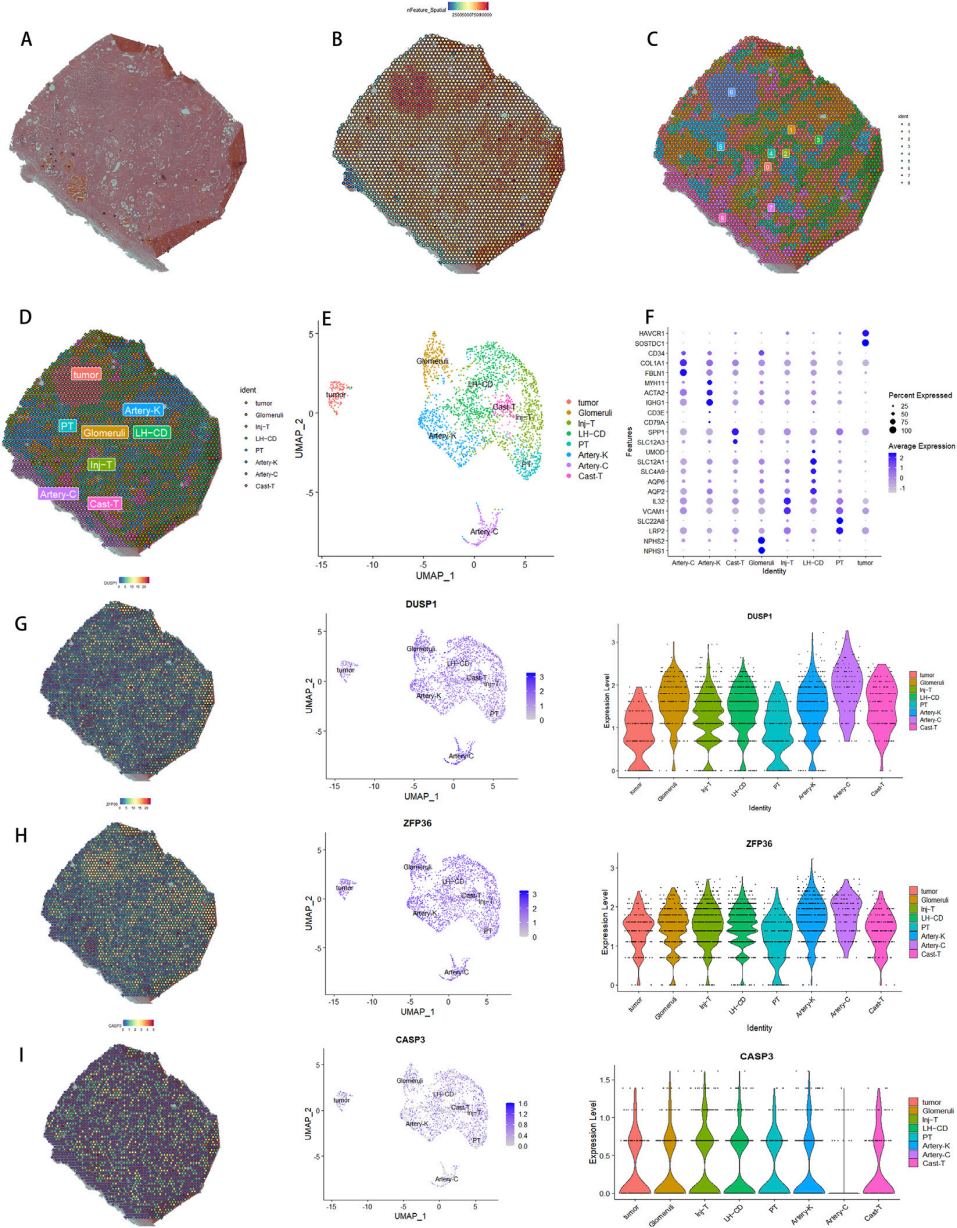
Spatial Transcriptomics (ST) Deconvolution Confirming the Local Morphological Archetype of Hypoxic Injury. **(A)** High-resolution histological H&E staining image of a representative human DKD kidney biopsy section. **(B,C)** Spatial transcriptomic feature processing. The quantitative capture heatmap **(B)** profiles 18,085 genes across 2,802 spatial sampling points (spots), with spatial dimensionality reduction (t-SNE/UMAP) mapped in **(C)**. **(D)** Spatial tissue cluster map annotated according to histological architectures (Glomerulus, Proximal Tubule, Distal Tubule, LOH-CD axis) via CARD deconvolution. **(E,F)** Parallel alignment with single-cell data. Dimension reduction of reference single-cell datasets **(E)** and corresponding expression matrix of validated cell markers **(F)** used for spatial mapping. **(G–I)** *In situ* spatial expression overlay.

For spatial transcriptome, 18,085 genes,2802 sampling points of a kidney tissue section were included in the clustering. The data quality control was shown in Supplementary Figures S13A,B. The section could be divided into eight regions (Figures 7A,B), which were as follows: glomeruli, injured tubules (Inj−T), arteries within the capsule (Artery−C), loop of Henle and collecting ducts (LH−CD), Proximal tubules (PT), tubules with endoluminal protein casts (Cast-T), arteries within the renal parenchyma (Artery-K) and tumor (Figures 7C–F). DUSP1 and ZFP36 have low expression in LH−CD and are widely distributed in all regions, while CASP3 has a low distribution, which were consistent with results of snRNA-seq (Figures 7G–I). Top 10 representative markers for each region was shown in Supplementary Figure S13C. KEGG enrichment of those genes revealed the high expression of HIF-1 signalling pathway in injured tubules and proximal tubules (Supplementary Figure S13D).

### 3.8 HIR score for the prognosis of DKD based on United Kingdom biobank proteomics

Among 918 DKD patients, 63 (6.9%) developed renal composite endpoint, as shown in Supplementary Table 2. The flowchart of patients’ screening was shown in Supplementary Figure S14. The composite kidney outcome group (N = 139) was significantly older, had longer diabetes duration, lower eGFR, higher ACR and urea, and greater cystatin C than controls (N = 779). When stratified, the primary outcome subgroup (renal death, N = 127) mirrored these differences, with larger waist circumference and higher CRP. The secondary outcome subgroup (N = 16) exhibited the most severe renal impairment with lowest eGFR (47.7 mL/min/1.73 m^2^), highest urea (13.2 mmol/L), and highest cystatin C (1.78 mg/L).

**FIGURE 8:**
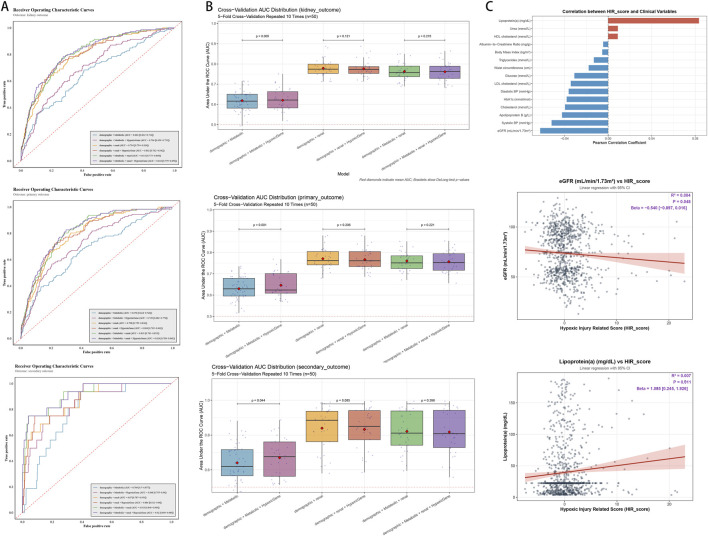
The significance of the HIR score based on United Kingdom Biobank proteomics in the prognosis of DKD. **(A)** ROC curve assesses the discriminatory efficacy of HIR score for different outcomes of DKD. The HIR score can significantly increase the AUC of each outcome in the basic demographic-metabolic model, but the marginal benefit narrows after including renal function indicators. **(B)** 5-fold cross-validation evaluates the robustness of incremental prediction of HIR score. This incremental value is highly reproducible and the DeLong test indicates that the HIR score has the most prominent independent predictive value in scenarios where renal indicators are lacking. **(C)** Correlation analysis of HIR score with clinical renal function and lipid metabolism indicators. The HIR score is negatively correlated with eGFR and positively correlated with Lipoprotein **(A)**. Kidney Outcomes (Composite): The overarching endpoint including both primary and secondary outcomes. Primary Outcomes (Renal Death): Specifically defined as death directly attributed to renal failure or associated kidney diseases. Secondary Outcomes: Includes clinical endpoints such as End-Stage Renal Disease (ESRD), initiation of Kidney Replacement Therapy (KRT), or a significant decline in eGFR (≥40% decrease from baseline). Demographics indicators include age, sex, education, TDI, ethnicity, and lifestyle factors (smoking, alcohol, sleep); metabolic indicators cover glycemic and lipid profiles (HbA1c, glucose, cholesterol, HDL-C, TG, LDL-C, Lp (a), ApoB), anthropometric and blood pressure measures (BMI, WC, DBP, SBP), diabetes duration, CRP, and concomitant medications; while renal indicators consist of eGFR, ACR, urea, cystatin C, albumin, and urinary electrolytes.

The multi-dimensional validation of HIR score in the proteinomics of United Kingdom Biobank for the prognosis of DKD was shown in Figure 8. In DKD patients, with the kidney composite outcome (kidney outcome), primary outcome (renal death, primary outcome), and secondary outcome (ESRD/KRT/eGFR decline ≥40%, secondary outcome) as endpoints, the predictive efficiency of 6 different models was tested. In the baseline model that only contained demographic and metabolic indicators, after adding the HIR score, the AUC of the three outcomes increased by approximately 0.043 (0.663–0.706), 0.053 (0.676–0.729), and 0.059 (0.789–0.848), respectively, and the 95% confidence intervals all shifted significantly upward. When the model already included eGFR, Urea, ACR, and other renal function indicators, the incremental contribution of adding the HIR score decreased (kidney: +0.008; primary: +0.006; secondary: +0.015) (Figure 8A). The AUC distribution obtained from 10 repeated 5-fold cross-validations (n = 50) showed that in the kidney and primary outcomes, the comparison between “demographic + metabolic” and “demographic + metabolic + HIR” was statistically significant (p = 0.005 and p = 0.001), and the secondary outcome also reached p = 0.044 (Figure 8B). The correlation heatmap showed that the HIR score has the strongest negative correlation with eGFR (the blue bars point to the left), and has a positive correlation with Lipoprotein(a) and Urea (Figure 8C).

### 3.9 *In Vivo* experimental validation of the HIR_score and core signatures

**FIGURE 9:**
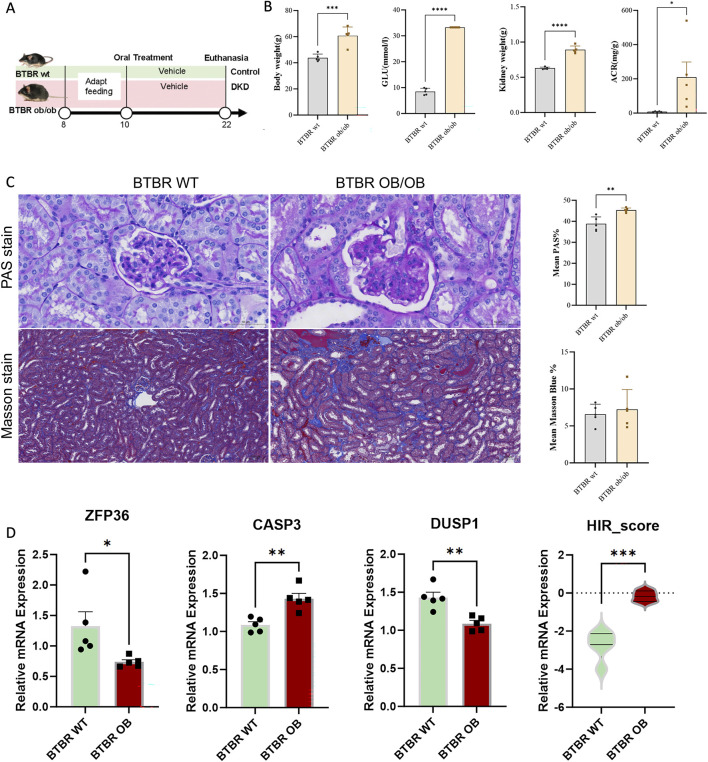
Experimental validation of the HIR score and its core component genes in BTBR ob/ob mice. **(A)** Schematic representation of the *in vivo* experimental timeline and treatment grouping utilizing 8-week-old male BTBR wild-type (WT) control mice and BTBR ob/ob diabetic kidney disease (DKD) mice. **(B)** Quantification of baseline physiological and clinical standard parameters at sacrifice, including body weight, blood glucose (GLU), kidney weight, and urinary albumin-to-creatinine ratio (ACR) (n = 5 per group). **(C)** Representative histopathological images of renal tissues from BTBR WT and BTBR ob/ob mice stained with Periodic Acid-Schiff (PAS) (upper panels, scale bar = 20 μm) and Masson’s trichrome (lower panels, scale bar = 50 μm), alongside corresponding semi-quantitative analyses evaluating the Mean PAS positive area (%) and Mean Masson Blue fibrotic area (%). **(D)** Quantitative real-time PCR (qRT-PCR) analysis documenting the relative mRNA expression profiles of core hypoxia injury-risk targets (ZFP36, CASP3, and DUSP1) normalized against GAPDH, together with the composite *in vivo* calculation of the integrated HIR_score signature. Data are presented as mean ± standard deviation (SD). Statistical significance was computed via Student’s t-test (*P < 0.05, **P < 0.01, ***P < 0.001, ****P < 0.0001).

To assess the biological validity and translational relevance of the computed computational signatures, we established an *in vivo* diabetic kidney disease (DKD) model using BTBR ob/ob mice alongside age-matched BTBR WT controls (Figure 9A). Phenotypically, 24-week-old BTBR ob/ob mice displayed profound diabetic nephropathy traits, characterized by significantly elevated body weight, blood glucose levels, kidney weight, and urinary albumin-to-creatinine ratios (ACR) compared to the WT cohort (Figure 9B). Histopathologically, PAS and Masson’s trichrome stainings demonstrated pronounced structural abnormalities, featuring prominent glomerular basement membrane thickening, expansion of the mesangial matrix, and extracellular matrix deposition in the diabetic group (Figure 9C). At the molecular transcript level, qRT-PCR analysis from pulverized renal tissues demonstrated that the expression profiles of the core prognostic candidates matched our algorithmic predictions. Specifically, the expression of the protective components ZFP36 and DUSP1 was significantly downregulated, whereas the pro-apoptotic executive vector CASP3 was markedly elevated in the DKD group. The calculated tissue-level HIR_score showed a highly significant and robust increase in the renal microenvironment of BTBR ob/ob mice (Figure 9D).

## 4 Discussion

Our study assessed the activity of 7 different types of hypoxic injury in DKD and further explored their activity in various kidney cells using snRNA-seq data. We also investigated the hypoxic expression patterns of hub genes in core cells and kidney regions. Additionally, we validated the relationships between these 7 types of hypoxic injury and immune infiltration, genes associated with microalbuminuria in DKD, and GFR. We constructed a HIR score based on 3 core hypoxic injury genes (CASP3, DUSP1, and ZFP36) using observational microarray transcriptome analysis. Both internal and external datasets showed that the HIR risk score was a powerful diagnostic indicator and a marker of disease severity in DKD patients. These results provided new insights into the role of HIR genes in the pathogenesis of DKD.

We found significant changes in the expression of Hypoxia, Autophagy, Ferroptosis, endoplasmic reticulum (ER) stress, and Apoptosis in the DKD group. The response to hypoxia is the initial and central aspect of cellular hypoxic injury. Hypoxia transcriptionally induces a robust set of genes controlled by HIFs but also a range of other transcription factors, including nuclear factor-κB (NF-κB) (Yang et al., 2025). The vast majority of O_2_ sensitive genes are in fact direct HIF targets. These genes at the cellular and organism level help adapt to diminishing levels of O_2_. However, persistent activation of hypoxia-induced genes can result in pathologies, including metabolic and kidney disease (Kaelin and Ratcliffe, 2008). Notably, our bulk tissue-level GSVA indicated a decreased overall activity of the hypoxia pathway in DKD, which seemingly conflicts with the paradigms of hypoxia-driven progression. This phenomenon might be rationalized by several factors. First, chronic adaptation and long-term evolutionary pressure in advanced DKD can prompt a feedback downregulation or exhaustion of classical hypoxia responsive machinery. Second, the prominent fibrotic remodeling and cellular dropout (e.g., specialized tubular segments) in bulk samples could cause a signal dilution effect, obscuring localized hypoxic stress, a notion supported by our single-cell and spatial evidence showing distinct cell-type specific hypoxic vulnerabilities.

In our study, we identified ZFP36, DUSP1, and CASP3 as new indicators of hypoxic injury. CASP3, or caspase-3, is positively correlated with DKD progression. It is involved in both hypoxia tolerance regulation and downstream apoptotic responses, which acts as the primary executioner caspase in apoptosis and is a key component of the cytotoxic T lymphocyte (CTL) killing mechanism (Atkinson et al., 1998). Under hypoxia, caspase-3 served as the main executioner caspase and lead to the morphological and biochemical changes (Jiang et al., 2025). Extensive research has demonstrated that CASP3 is a risk factor for the onset and progression of DKD. In animal experiments, Sekiko et al. found that caspase-3 was significantly upregulated in the renal tubular cells of diabetic mice (Taneda et al., 2010). Wen et al. observed that injecting caspase-3 inhibitors into streptozotocin-induced diabetic mice significantly alleviated albuminuria, improved renal function, and mitigated pathological changes (Burlaka, 2022). Clinical observations showed that in patients with type 1 diabetes and early diabetic nephropathy, increased caspase-3 levels were associated with a higher risk of DKD (Wen et al., 2020).

This study showed that ZFP36 and DUSP1 were significantly negatively correlated with DKD progression. The zinc finger protein 36 homolog (ZFP36), also known as tristetraprolin (TTP), is a protein that plays a regulatory role in hypoxic injury by stabilizing HIF-1α mRNA. The ZFP36 protein directly binds to the 3′-UTR of HIF-1α mRNA, thereby reducing the stability of HIF-1α mRNA. It plays a physiological role in regulating cellular adaptation and apoptosis under prolonged hypoxia (Kim et al., 2010; Chamboredon et al., 2011). A decrease in ZFP36 has been shown to be a risk factor for the progression of DKD (Luo et al., 2023; Tang et al., 2024; Liu et al., 2015). DUSP1, or Dual Specificity Phosphatase 1, is an important downstream regulator of the hypoxia tolerance response. In hypoxic conditions, DUSP1 is upregulated through an HIF-1α-dependent mechanism. It helps cells adapt to low oxygen environments by negatively regulating the MAP kinase signaling pathway and dephosphorylating tyrosine and threonine/serine residues on MAP kinases such as ERK, JNK, and p38 MAPK. This lowers the apoptotic threshold after hypoxic injury, enhancing cell survival (Rininger et al., 2012). A decrease in DUSP1 has been shown to be a risk factor for the progression of DKD (Sheng et al., 2019; Lu et al., 2021).

Single-cell transcriptomics revealed that ZFP36 and DUSP1 were significantly lower expressed in Loop of Henle cells of DKD, which have a high HIR risk score simultaneously. These cells communicated with most other cell types via the EGF pathway. This aligned with previous research findings. The Loop of Henle (LOH) is a critical segment of the kidney involved in reabsorption. Its ascending limb, particularly the thick ascending limb (TAL), efficiently reabsorbs Na^+^, K^+^, and Cl^−^, creating a hyperosmotic environment in the renal medulla, facilitating water reabsorption in the collecting duct (Hebert and Andreoli, 1984). Due to its deep medullary location, LOH cells are especially sensitive to hypoxic conditions. Hypoxia can lead to metabolic disturbances and dysfunction in these cells, triggering a cascade of hypoxic injury in DKD (Brezis et al., 1988). Necrosis of the TAL in isolated perfused kidneys has been considered an important model of renal hypoxia due to its pathological sensitivity to hypoxic injury (Shanley and Johnson, 1989). P. J. Ratcliffe and colleagues established a controlled hypoxia model in perfused rat kidneys to compare the extent of damage between glomerular and tubular cells. They found that the medullary TAL was more sensitive to hypoxic injury compared to other renal segments (Ratcliffe et al., 1988). This characteristic likely explained why these cells uniquely express hub genes associated with hypoxic injury. Notably, the spatial map revealed a striking concordance between regional hypoxia and the spatial distribution of the HIR signature. Within the LH-CD region-anatomically confined to the hyperosmotic renal medulla-marked suppression of DUSP1 and ZFP36 coincides with strong induction of the HIF-1 signalling pathway, confirming that these tubulo-medullary segments are the epicentre of hypoxic stress in DKD.

To bridge the gap between tissue-level hypoxic signatures and clinical applicability, we further validated the prognostic value of the HIR score using large-scale proteomic data from the United Kingdom Biobank. The HIR score significantly improved the discrimination of kidney outcomes, renal death, and secondary endpoints over baseline demographic and metabolic models, with AUC increments of approximately 0.04–0.06 (P < 0.05 via DeLong test, robust across 10-times repeated 5-fold cross-validation). However, in alignment with the realistic constraints of modern renal diagnostics, the incremental predictive gain of the HIR score diminished substantially (approx. +0.006 to +0.015) after the fully adjusted inclusion of conventional clinical pillars (eGFR, urea, and ACR). This marked attenuation honestly indicates a significant informational overlap, suggesting that our proteomic signature shares common pathological covariance with standard functional markers of renal decline. Consequently, the core utility of the HIR score may not lie in completely outperforming or displacing existing non-invasive laboratory standards. Instead, its independent clinical value resides heavily in providing granular, patient-specific mechanistic insights, serving as a molecular lens that reads out cellular-level hypoxic stress patterns and tissue-level microenvironment remodeling, which cannot be captured by routine creatinine or albuminuria surveillance. Phenotypically, the HIR score correlated negatively with eGFR and positively with lipoprotein(a), reinforcing the known interplay between hypoxia-driven tubulointerstitial damage and dyslipidemia in DKD progression. These findings present the HIR signature not as a mere algorithmic replacement for standard tests, but as a pathologically informative risk-stratification tool for developing non-invasive blood- or urine-based mechanistic assays in routine clinical practice.

In the future, the HIR score will be integrated into precision clinical workflows as a three-tiered management pipeline: first, serving as an early-stage “molecular biopsy” to identify high-risk fast-progressors before irreversible eGFR decline occurs, enabling timely, targeted interventions (e.g., SGLT2 inhibitors or HIF stabilizers); second, transitioning into non-invasive liquid biopsy formats, specifically plasma multiplex digital-ELISAs and urine extracellular vesicle mass spectrometry (PRM) assays, to circumvent the procedural constraints of repeat tissue biopsies; and third, functioning as a dynamic companion diagnostic tool to track real-time therapeutic efficacy and guide proactive, individualized treatment adjustments during routine outpatient follow-ups.

This study has certain limitations. First, while our study successfully cross-validated the diagnostic accuracy and directional expression of ZFP36, DUSP1, and CASP3 within the heterogeneous microenvironment of BTBR ob/ob diabetic kidneys, the direct intracellular causalities governed by these vectors remain to be fully characterized. Future mechanistic inquiries are warranted to bridge this gap. We propose to establish a high-glucose combined with hypoxia microenvironment in human renal proximal tubular epithelial cells (HK-2). By deploying stable gain-of-function (plasmid overexpression) and loss-of-function (siRNA/shRNA knockdown) assays for ZFP36, DUSP1, and CASP3 respectively, their precise causal contributions can be delineated.

Second, although we have successfully projected our tissue-derived HIR score onto circulation profiles and verified its prognostic robustness using the large-scale retrospective United Kingdom Biobank proteomic cohort, certain clinical translation barriers remain. The current validation relies on existing public proteomic platforms rather than a customized, clinic-ready diagnostic assay. To further bridge this gap, true non-invasive deployment requires transitioning from retrospective database mining to targeted clinical validation. Third, risk prediction for DKD involved not only gene expression but also methylation modifications, proteomic features, and post-transcriptional regulation. To enhance the reliability of the data, integrating multi-omics approaches would be beneficial for refining the model.

In summary, we identified five key hypoxic injury pathways in DKD development and progression. The HIR score, based on hypoxic injury genes, effectively distinguished between normal controls and DKD patients and predicts renal function changes and kidney events. This provided valuable insights for clinical evaluation and management of DKD.

## Data Availability

Publicly available datasets were analyzed in this study. This data can be found here: GEO at https://www.ncbi.nlm.nih.gov/gds: GSE96804, GSE30528, GSE30529, GSE104954, GSE99339, GSE131882, GSE195460, GSE261545.
