# Supplementary material for: Decoding the hypoxic injury landscape and hypoxic risk model construction in diabetic kidney disease: a multi-omics study
Source: Front Bioinform. 2026 Aug 5;6:1894439. doi: 10.3389/fbinf.2026.1894439 (PMC13486302; doi:10.3389/fbinf.2026.1894439)
Supplement: Supplementary file 1 [file DataSheet1.pdf]

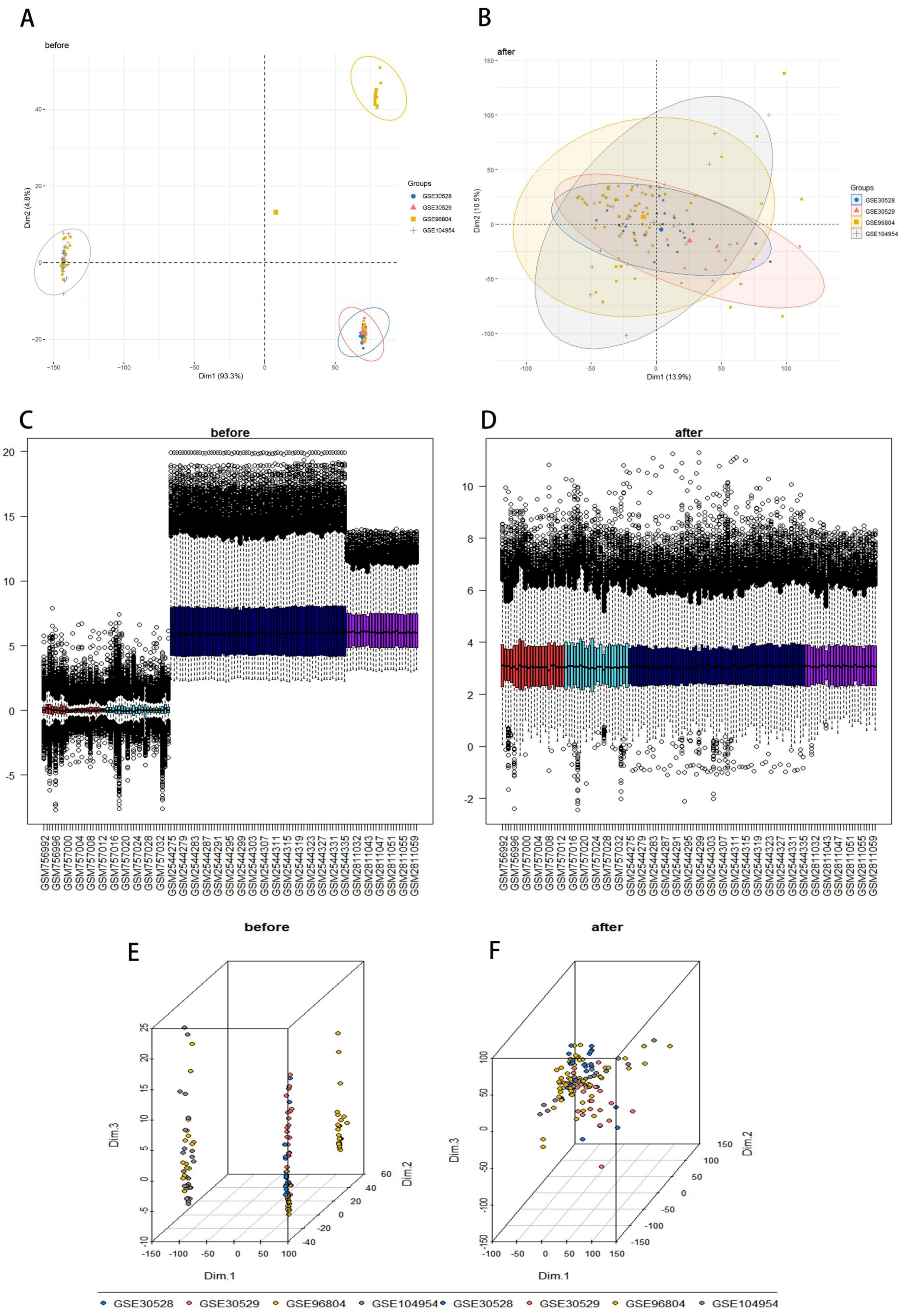

Supplementary Figure 1. Data preprocessing. (A, B) PCA of datasets before (A) and after (B) data processing. (C, D) Box plots of datasets before (C) and after (D) data processing. (E, F) 3D projection plots of datasets before (E) and after (F) data processing.According to parallel Principal Variance Component Analysis (PVCA) models, the total transcriptomic variance partition directly attributed to the technical

"batch factor" collapsed from 58.4% (Before) to 1.6%(After), whereas the meaningful biological variance component was robustly preserved and enhanced to 76.9%, demonstrating optimal data harmonization.

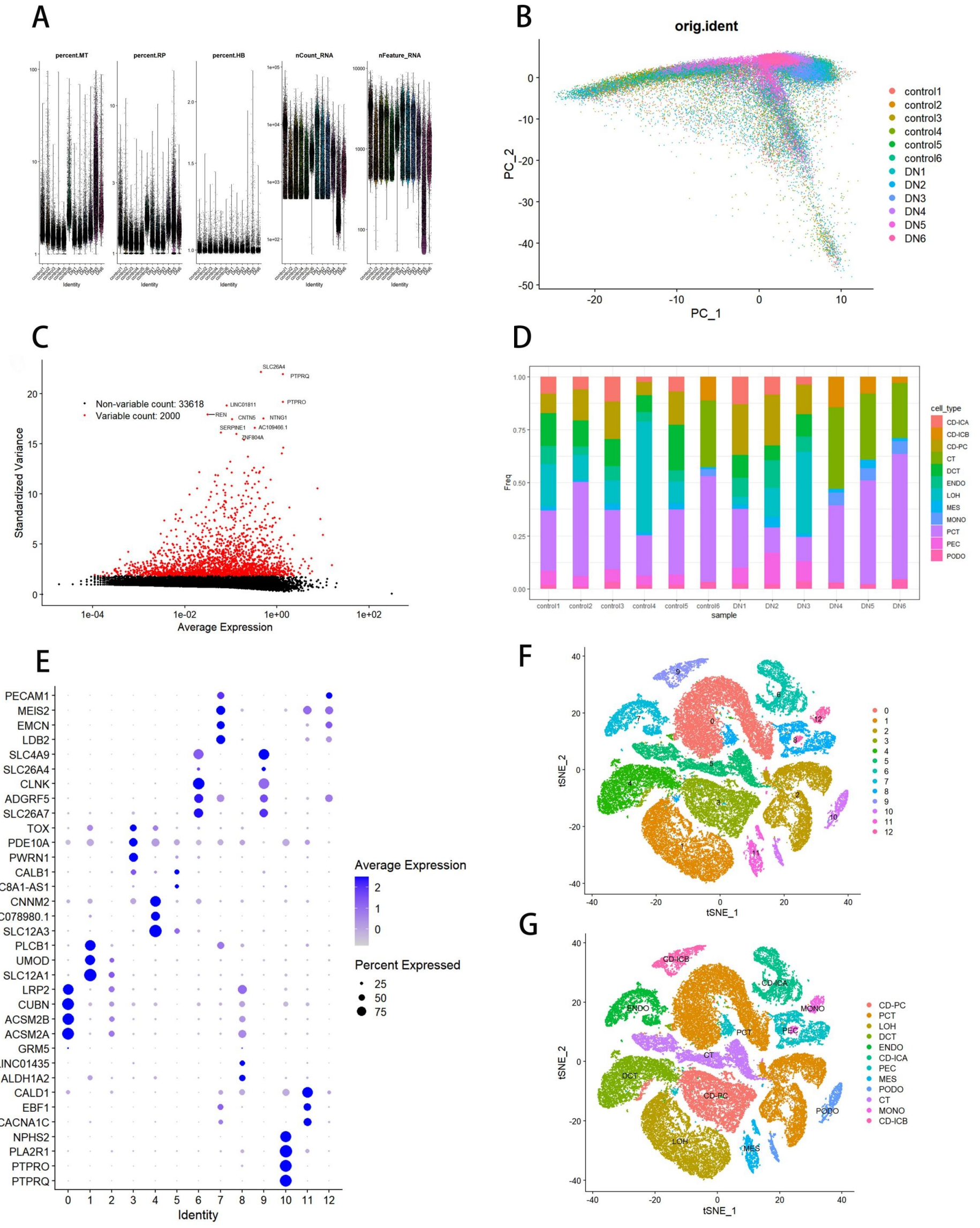

Supplementary Figure 2 The quality control and annotation results of the DKD single cell transcriptome data. Cell line: kidney cortex. (A) Quality control in defining the ratio of mitochondrial, ribosome and hemoglobin genes. (B) Batch effect was eliminated after standardization in the DN (6 samples) and control groups (6 samples, healthy donater). (C) The top 2,000 highly variable genes were shown. (D) The cell

types were roughly the same between the samples. (E) The correlation between cell markers and clusters. (F)(G) Cell annotation: mesenchymal cell(MES), glomerular parietal epithelial cell(PEC), proximal convoluted tubular cell(PCT), Loop of Henle cell(LOH), distal convoluted tubular cell(DCT), convoluted tubular cell(CT), collecting duct-principal cell(CD-PC), collecting duct-intercalated cell type A(CD-ICA), collecting duct-intercalated cell type B(CD-ICB), podocyte(PODO) , endothelia cell(ENDO) and monocyte(MONO).

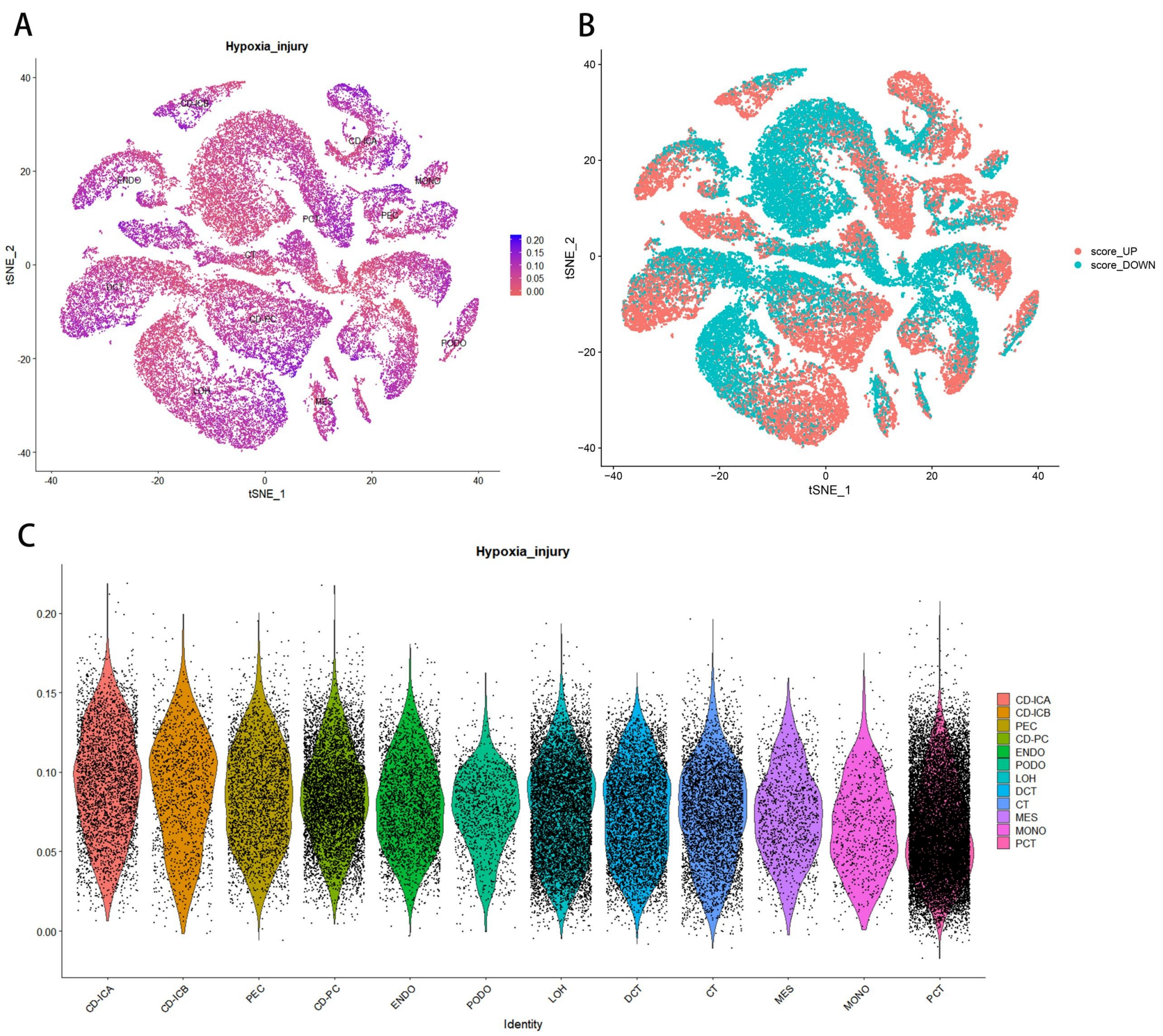

Supplementary Figure 3. Expression of HIR gene set in different renal cells in DKD patients. (A) Thr AUCell analysis on a HIR gene set with 12 types of renal cells. (B) The cell distribution based on median score of HIR gene set. (C)The sequencing of AUCell scores on all cell types.

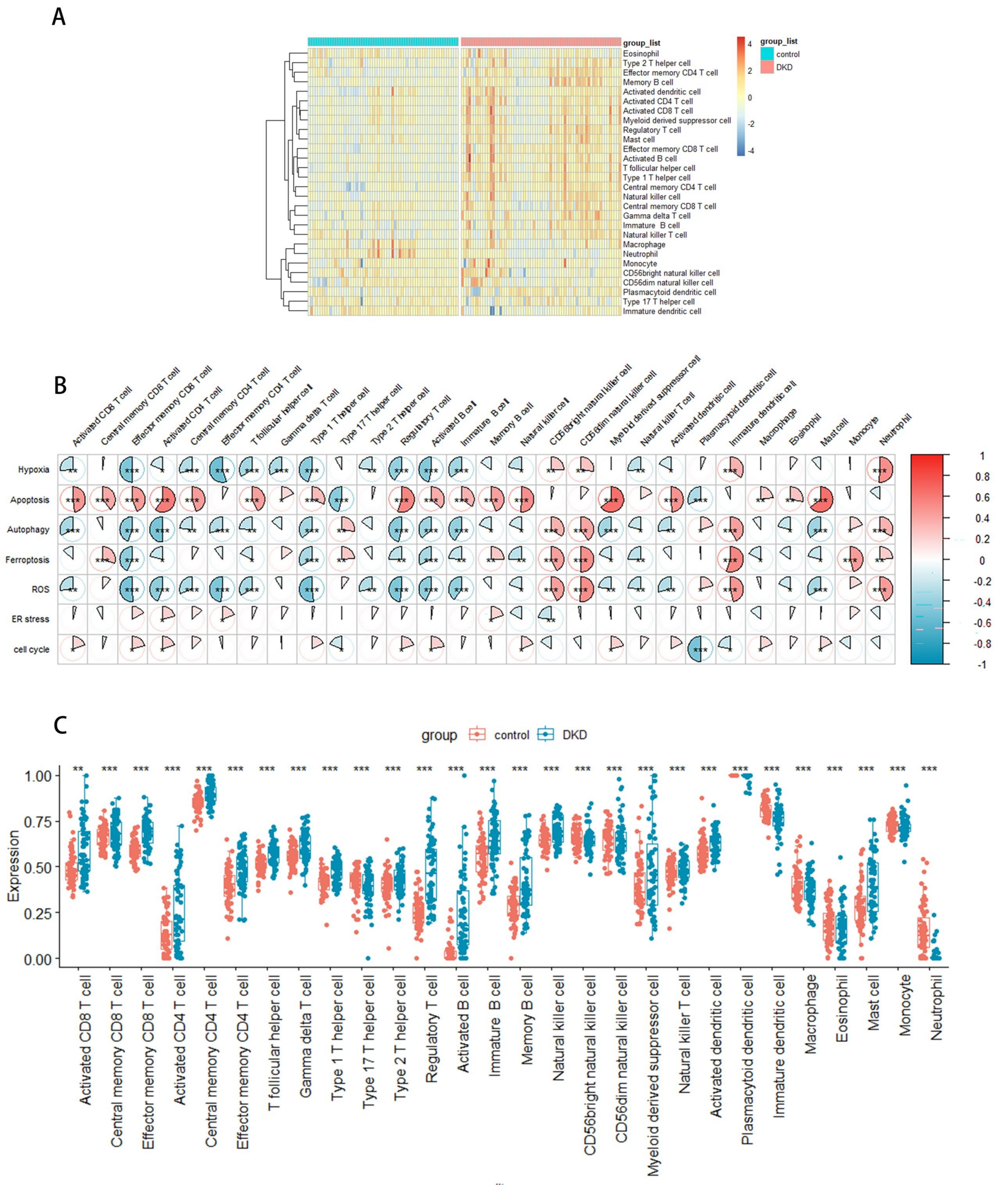

Supplementary Figure 4. Evaluation of immune cell infiltration. (A, C) The score of immune cells was detected by ssGSEA analysis in control and DKD groups. (B) Correlation analysis between the 7 HIR GSVA scores and different immune cells. *p < 0.05, **p < 0.01, ***p < 0.001.

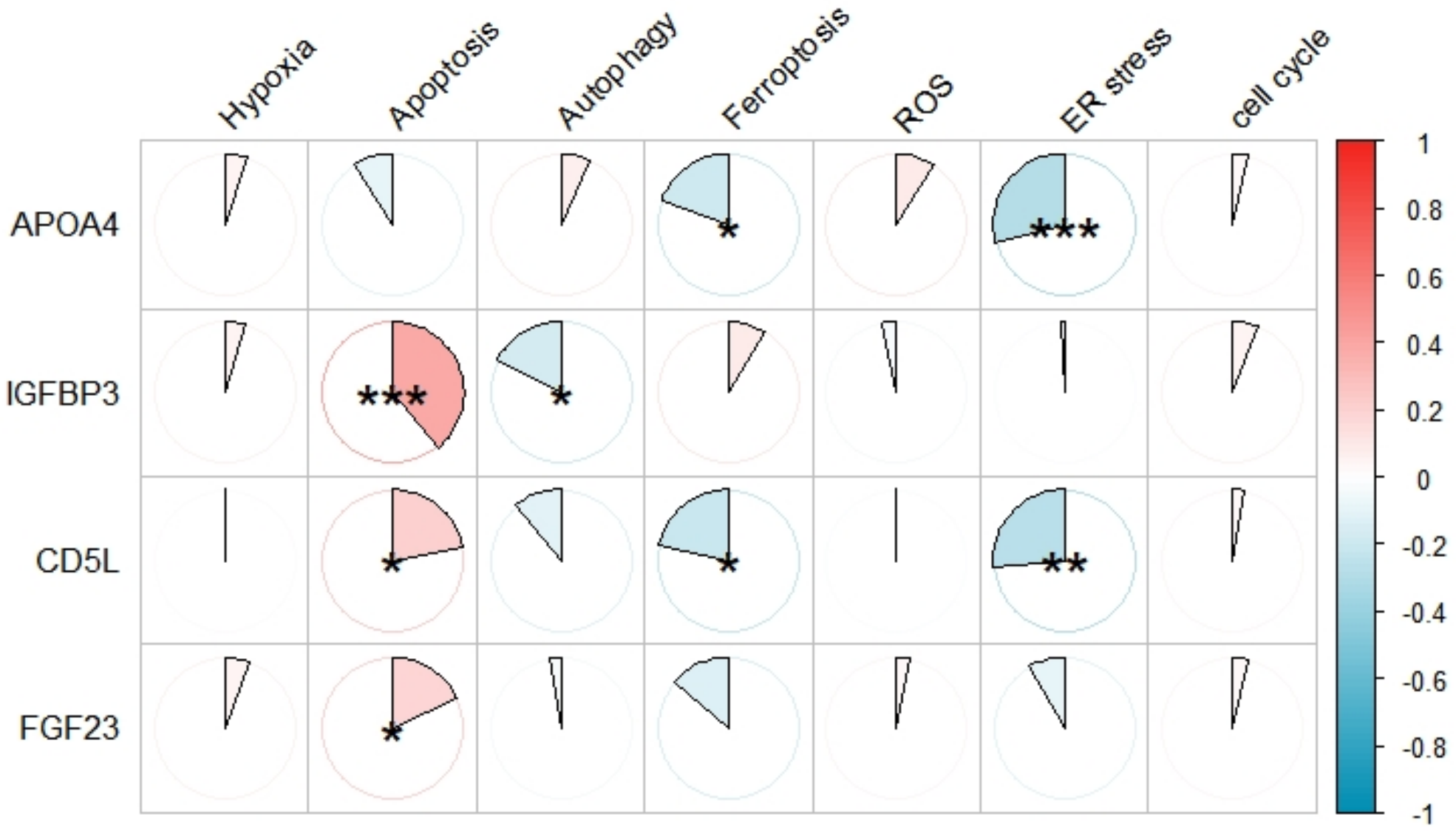

Supplementary Figure 5. Correlation analysis between the HIR geneset GSVA scores and renal fibrotic markers (FN1, COL1A2, COL1A1, and ACTA2). *$p < 0.05$, **$p < 0.01$, ***$p < 0.001$.

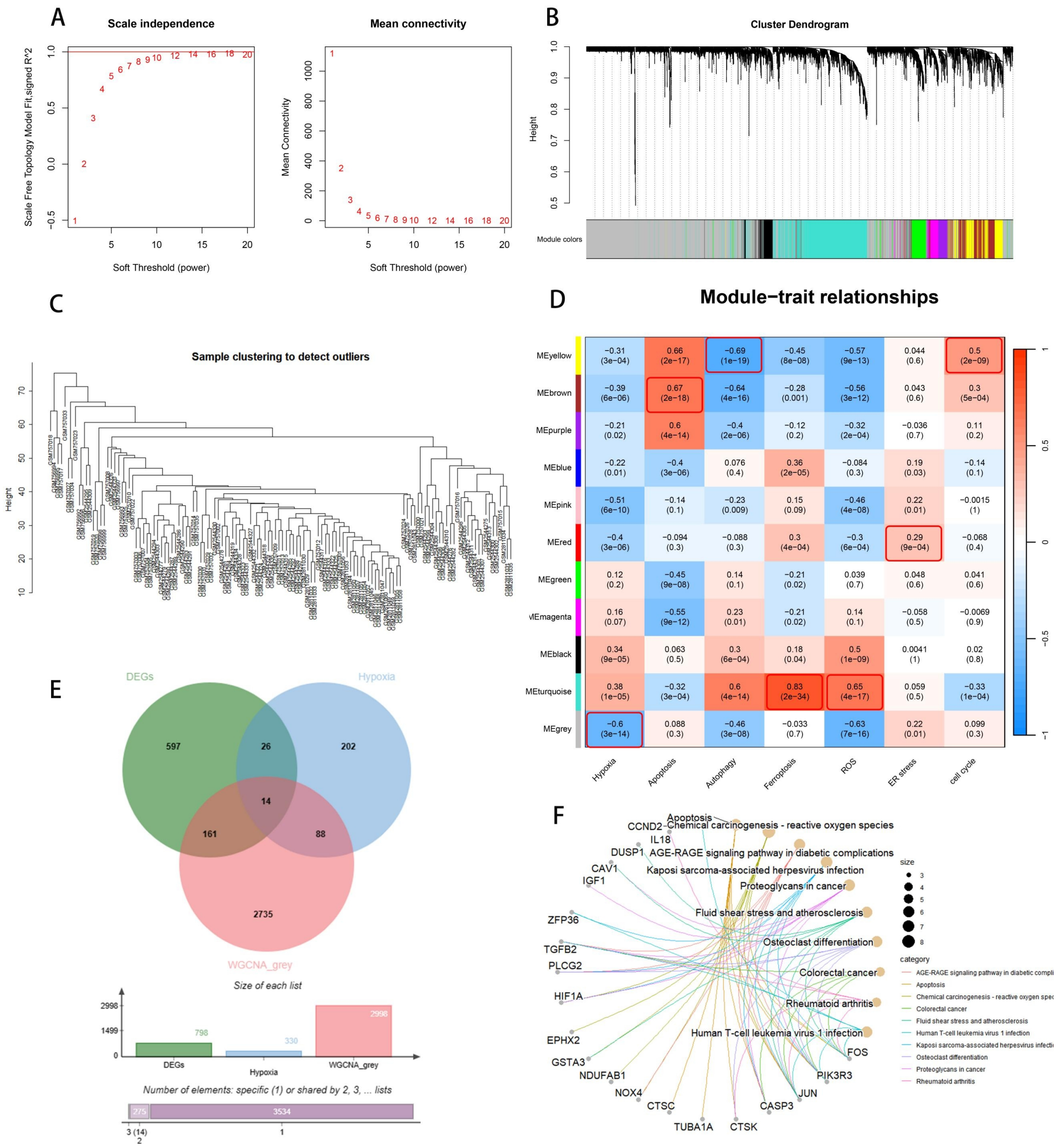

Supplementary Figure 6. Identification of the module closely related to HIR geneset in DKD by WGCNA. (A) Scale-free exponent and average connectivity for each soft threshold. (B) Dendrogram of gene clusters, with different colors representing different modules. (C) The clustering tree for all samples. (D) Heatmap of the correlations between modules and GSVA scores of 7 HIR pathways. (E) Venn diagram of the intersection of grey module genes, DEGs and hypoxia geneset. (F) KEGG enrichment analyses based on the intersection gene (7 set).

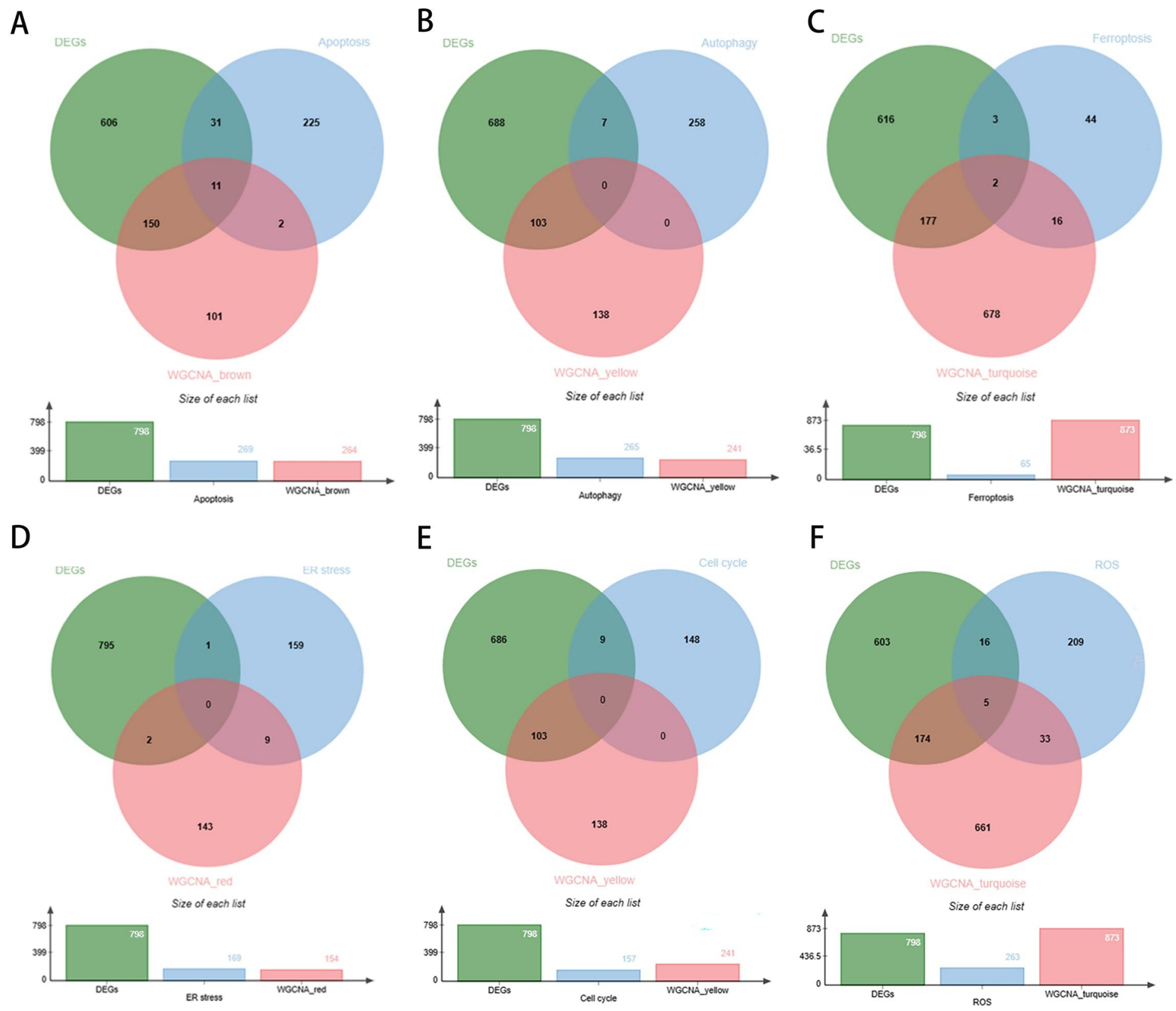

Supplementary Figure 7. Identification of other 6 key HIR genes. (A) Venn diagram analysis of intersection between brown module genes, DEGs and Apoptosis gene set. (B) intersection between yellow module genes, DEGs and Autophagy gene set. (C) turquoise, Ferroptosis and DEGs. (D) red, ER stress and DEGs. (E) yellow, Cell cycle and DEGs. (F) turquoise, ROS and DEGs.

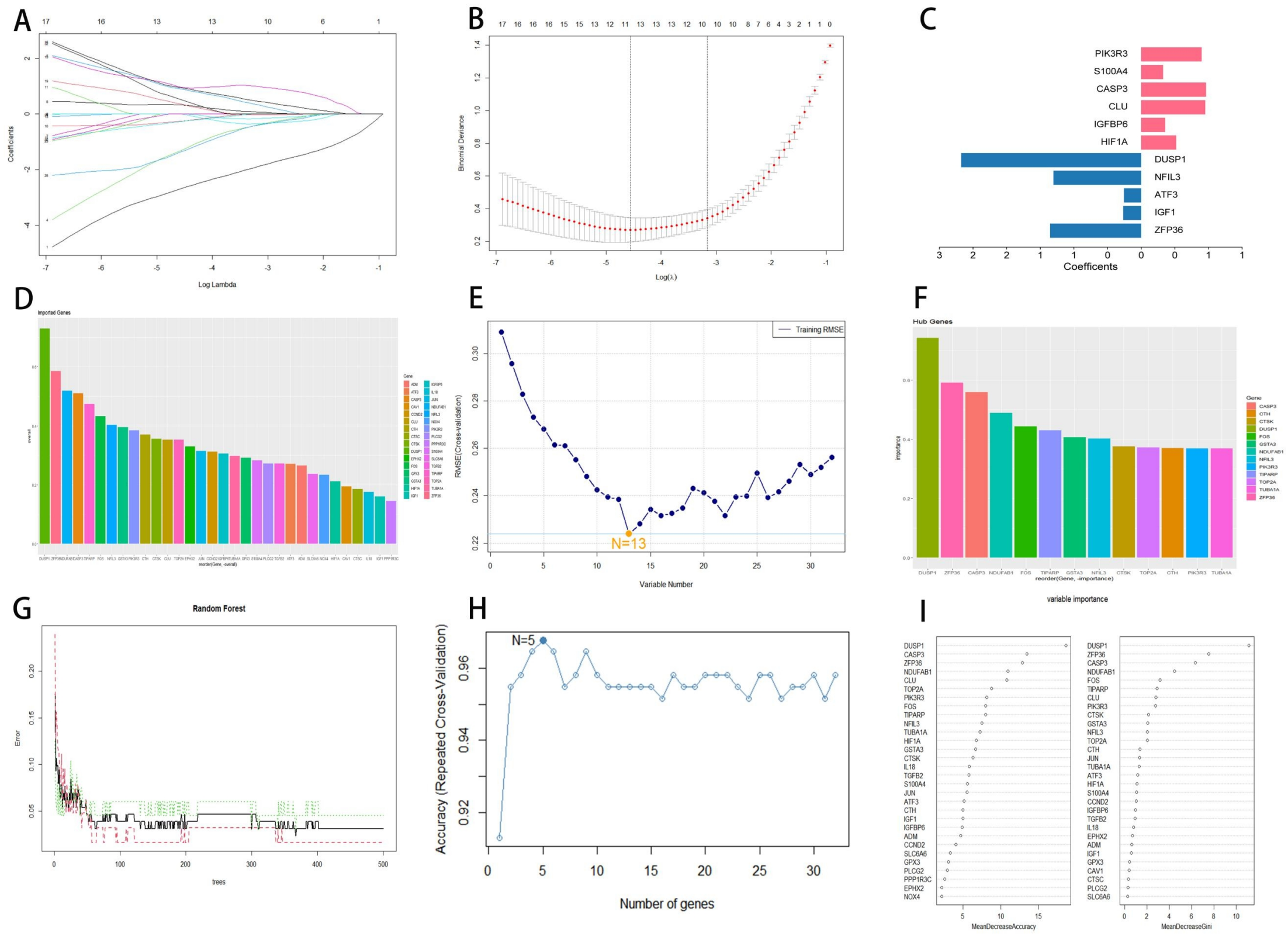

Supplementary Figure 8. Screening of hub gene by machine learning. (A,B,C)LASSO in DKD dataset: 11 genes have passed the screen. (D,E,F) SVM-RFE in DKD dataset: When n=13, the root mean square error (RMSE) was the lowest and the model performed best. (G,H,I) RRF in DKD dataset: When n=5, the diagnostic accuracy was the highest.

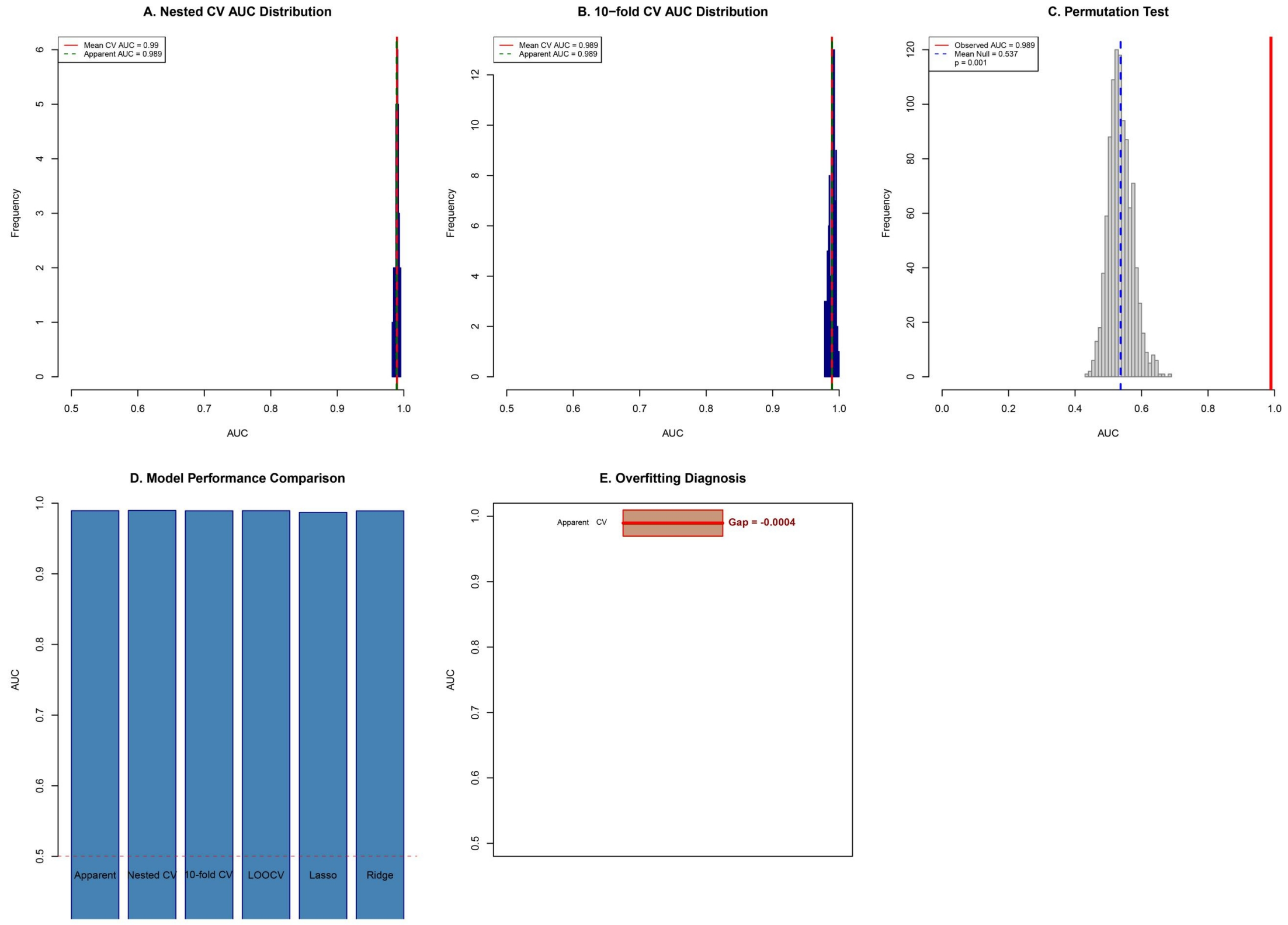

Supplementary Figure 9 Comprehensive validation of the HIR score for DKD diagnosis. (A) Distribution of AUC values from nested cross-validation (5-fold outer, 3-fold inner, 50 repeats). The red line indicates the mean nested CV AUC (0.9897), and the green dashed line indicates the apparent AUC (0.9893). (B) Distribution of AUC values from 10-fold cross-validation (100 repeats). (C) Permutation test showing the null distribution of AUC values from 1,000 random permutations of group labels. The red line indicates the observed AUC (0.9893, empirical p = 0.001). (D) Comparison of AUC values across different validation strategies. (E) Overfitting diagnosis showing the minimal gap between apparent AUC and nested CV AUC (overfitting gap = -0.0004).

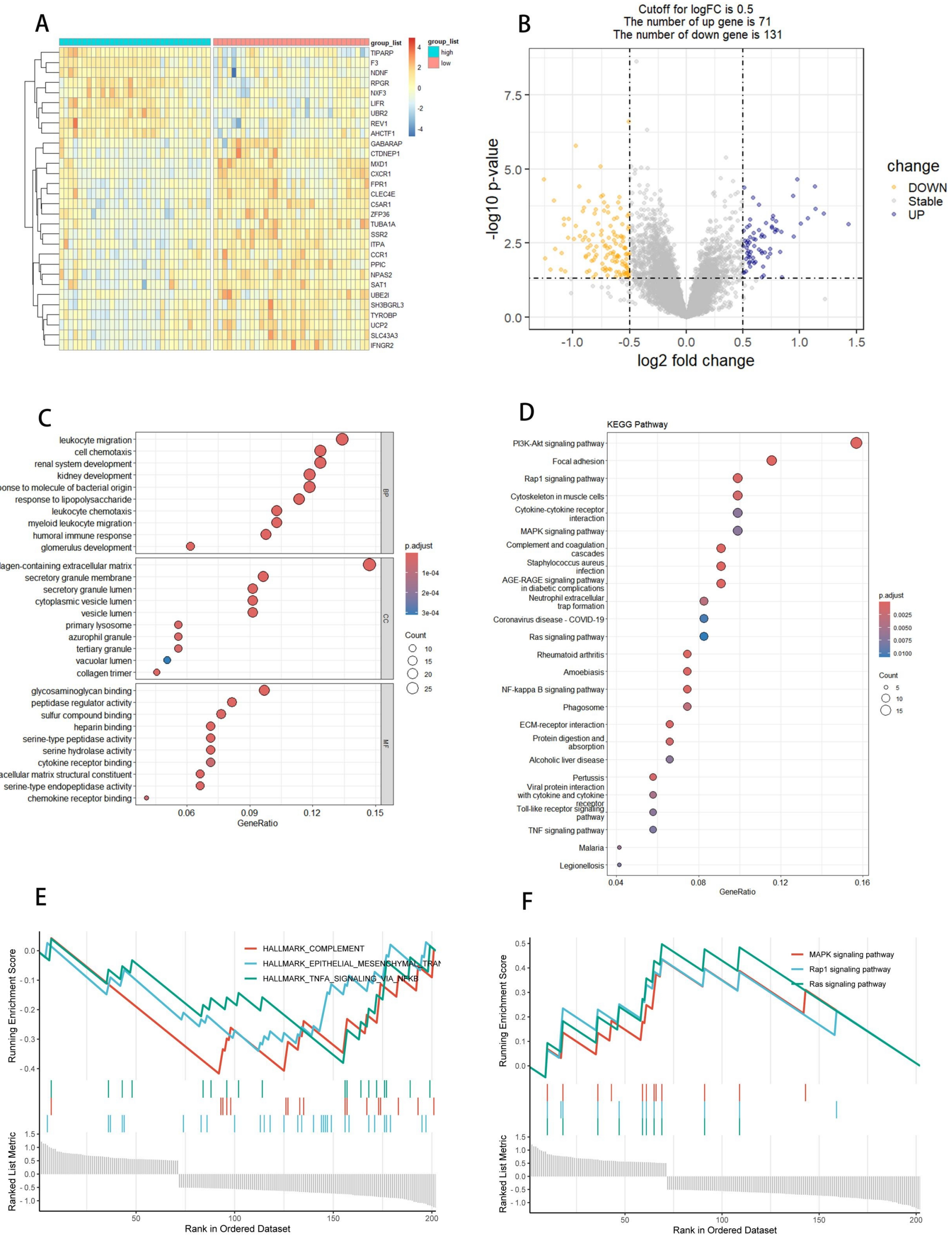

Supplementary Figure 10. Functional enrichment analysis of DEGs in the low-risk and high-risk groups. (A, B) Heatmap and the volcano plot of the DEGs between the low-risk and high-risk groups. (C) GO terms enriched in the DEGs between the low-risk and high-risk groups. (D) KEGG pathway between the low-risk and high-risk groups. (E, F) GSEA analysis of pathways in hallmark and positional gene sets.

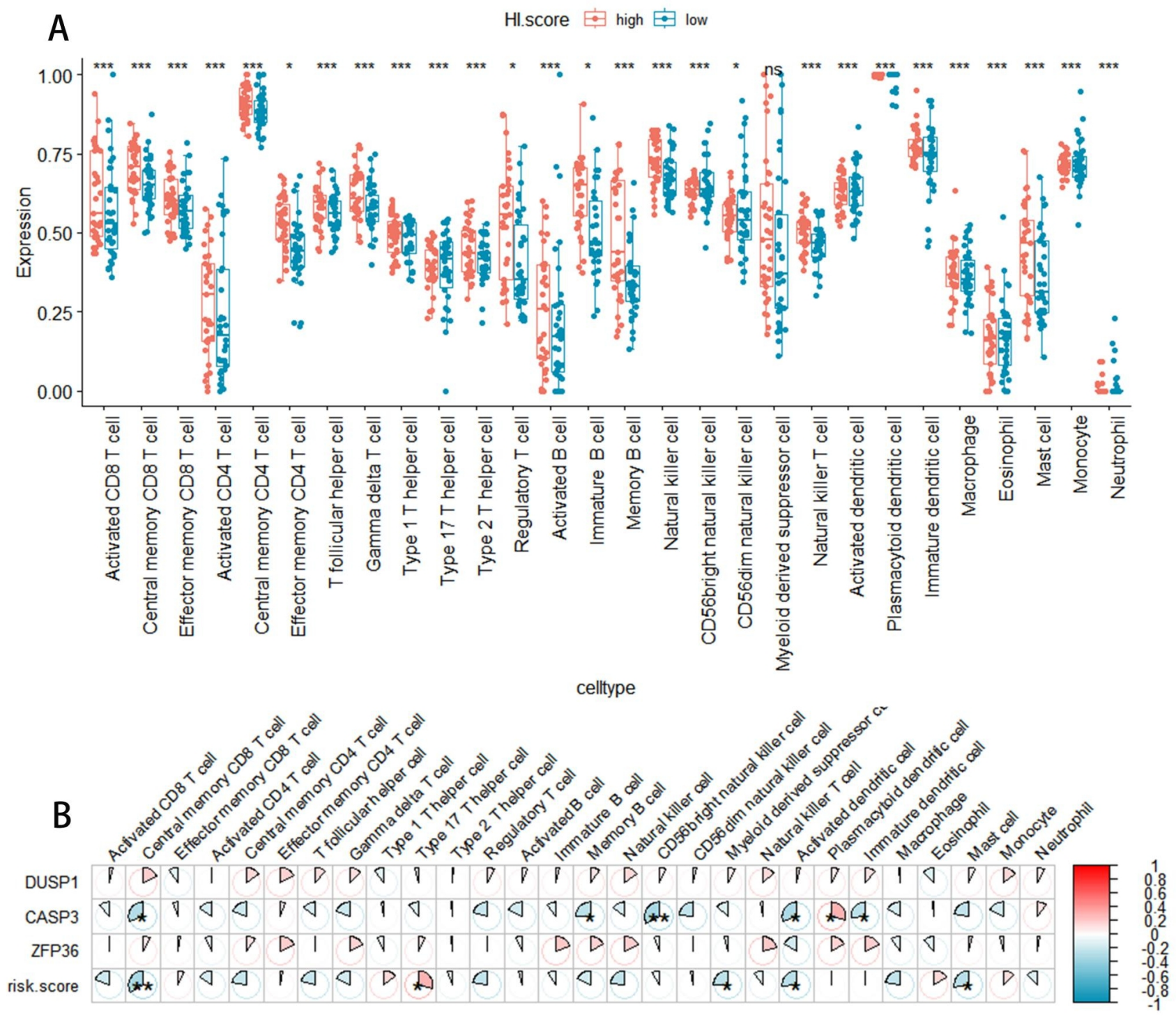

Supplementary Figure 11. Evaluation of immune cell infiltration in the low-risk and high-risk groups. (A) The score of immune cells was detected by ssGSEA analysis in the low-risk and high-risk groups. (B) Correlation analysis between the HIR risk score and 3 core death genes and different immune cells. *p < 0.05, **p < 0.01, ***p < 0.001.

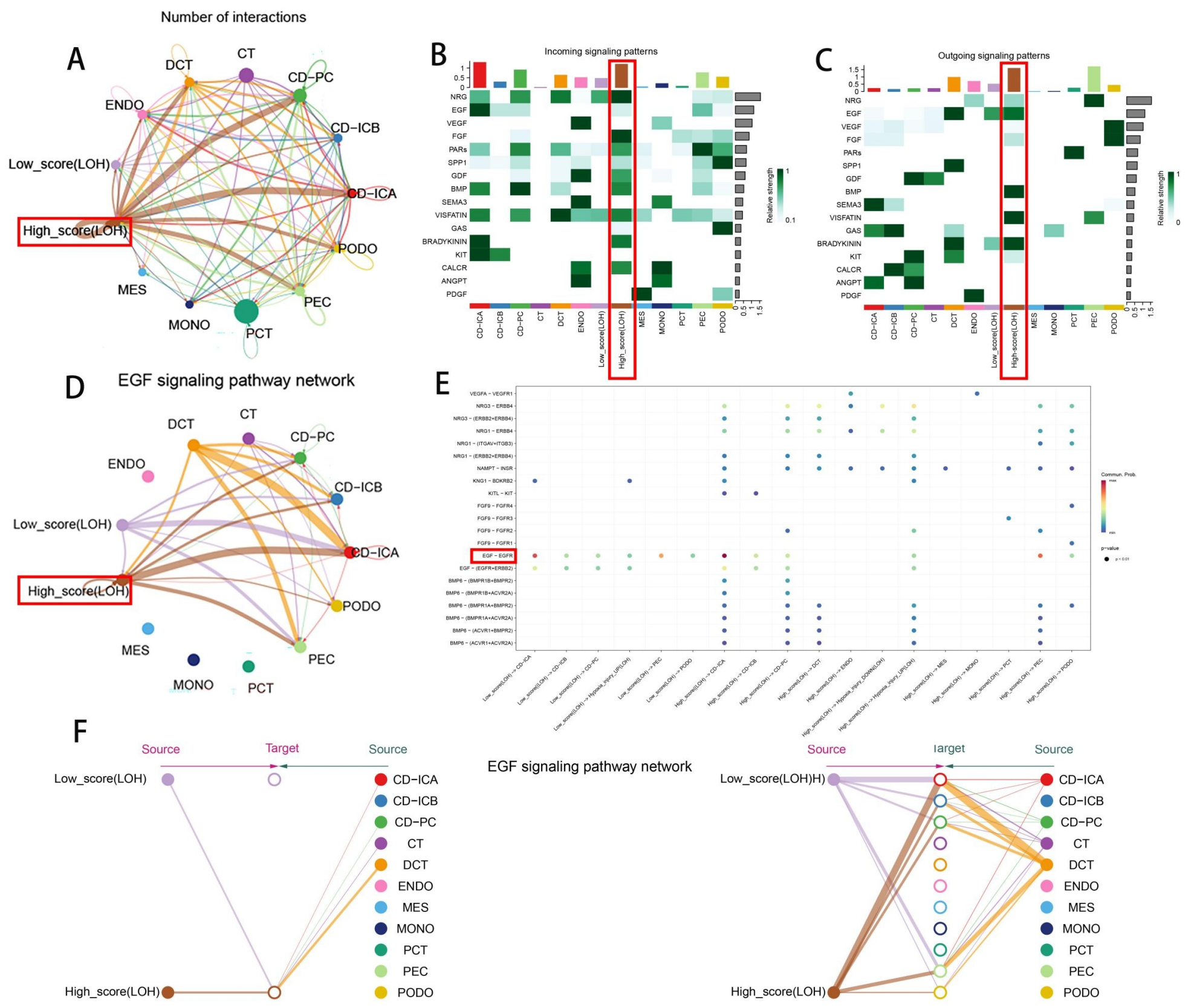

Supplementary Figure 12. Communication modes of LOH. (A) Communication between the high-HIR risk subgroup of LOH cells and other cell types. (B,C) Signaling patterns of cells. (D,E,F) LOH cells with high HIR risk scores communicated with most other cell types through the EGF pathway.

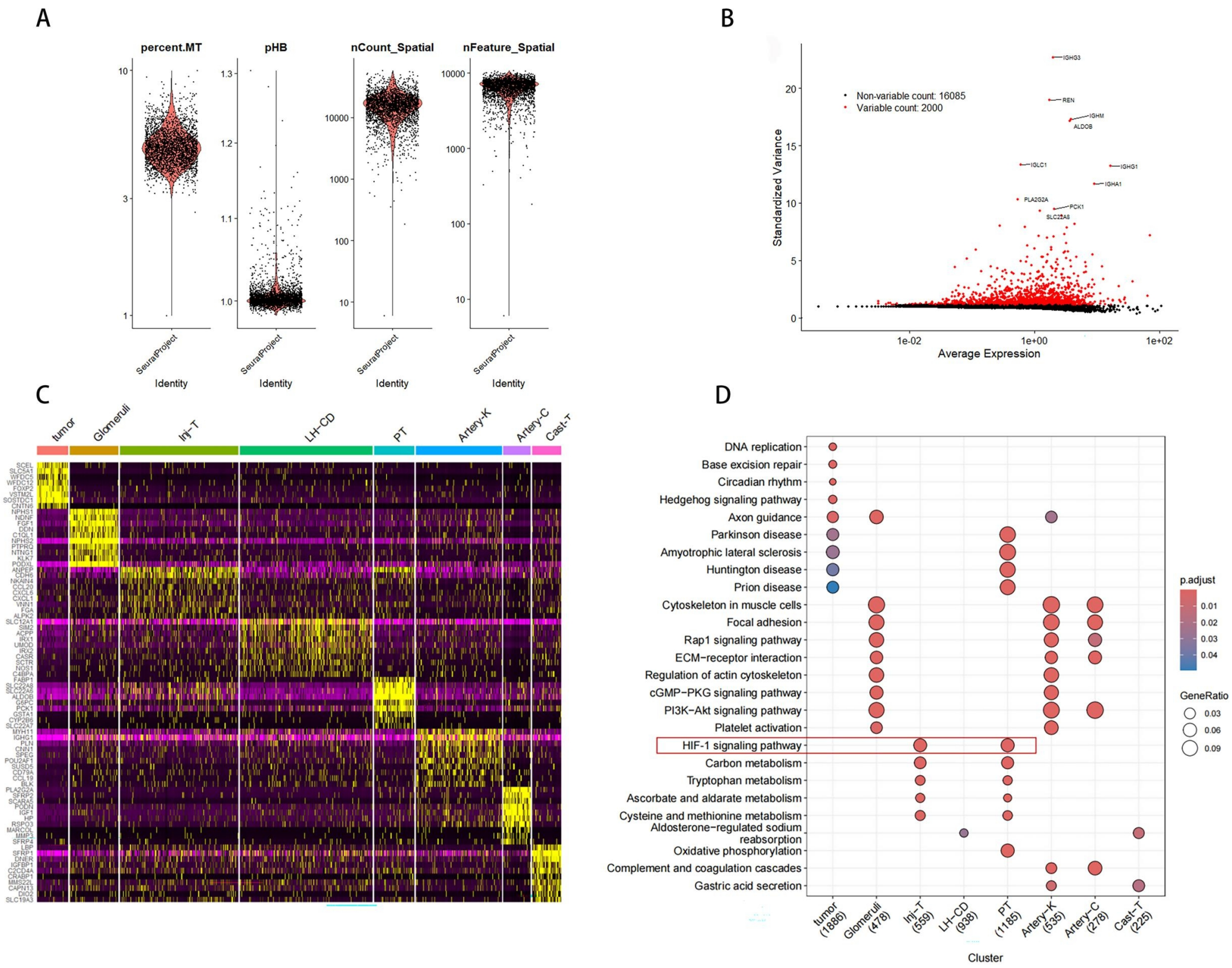

Supplementary Figure 13. Quality control and pathway enrichment for the spatial transcriptome. (A) Quality control after defining the ratio of mitochondrial and hemoglobin genes. (B) The top 2,000 highly variable genes were shown. **(C)**Top 10 representative markers for each region.(D)KEGG enrichment of representative genes in each region, with HIF-1 pathway activating in injury tubule region.

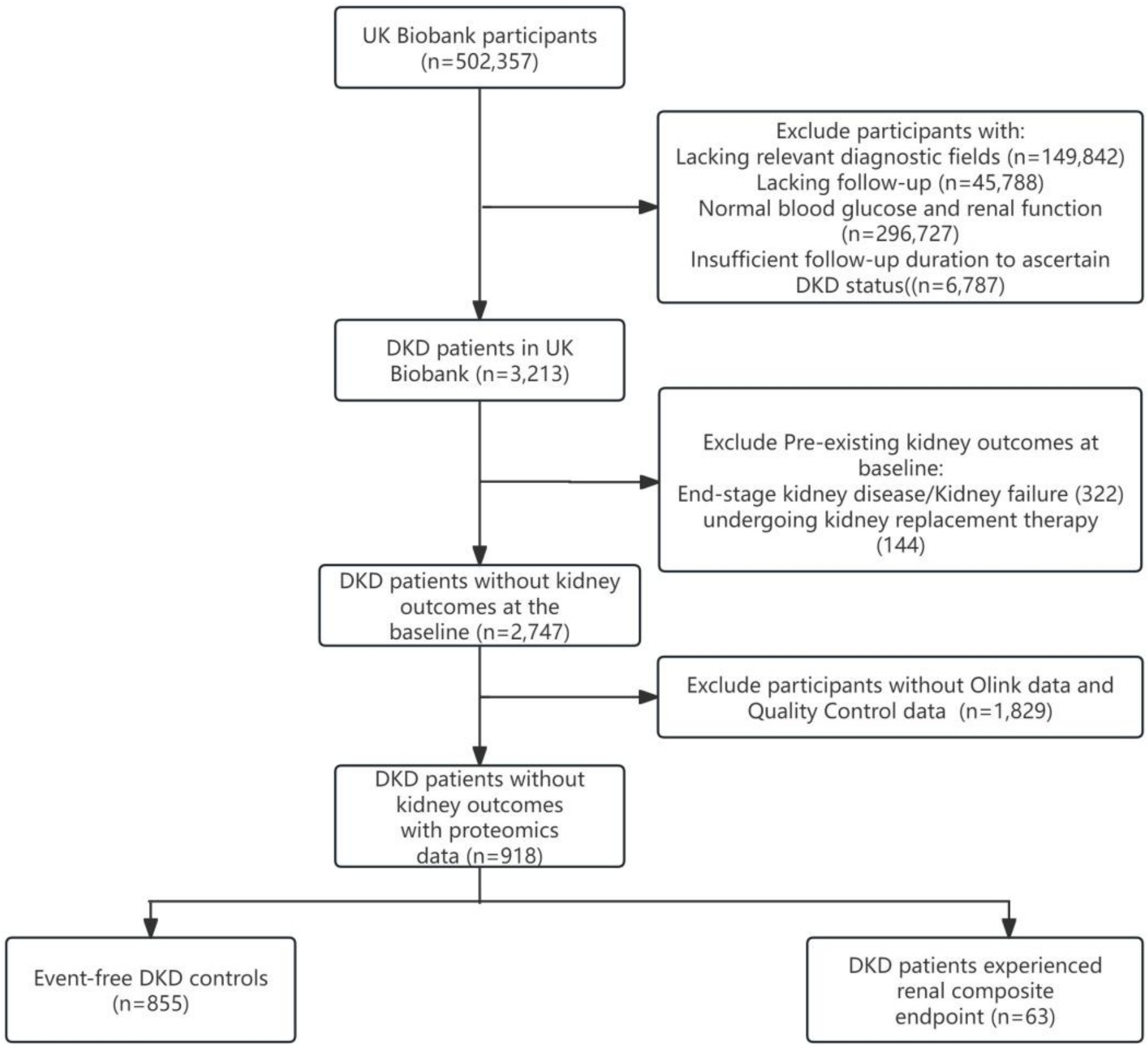

Supplementary Figure 14 Flowchart of participant selection

Supplementary Table 1 Characteristic of dataset included in the analysis

Dataset	Database	Platform	Sample	Tissue
GSE96804	GEO	GPL17586	41 cases of DKD, 20 cases of control	Glomerulus
GSE30528	GEO	GPL571	9 cases of DKD, 13 cases of control	Glomerulus
GSE30529	GEO	GPL571	10 cases of DKD, 12 cases of control	Tubules
GSE104954	GEO	GPL24120	7 cases of DKD, 18 cases of control	Tubules
	GEO	GPL19184 GPL19109	14 cases of DKD, 14 cases of control	Kidney biopsies
GSE131882	GEO-Single Cell Transcriptomic	GPL24676	3 cases of early DKD, 3 cases of controls	kidney tissue
GSE195460	GEO-Single Cell Transcriptomic	GPL24676	3 cases of early DKD, 3 cases of controls	kidney tissue
GSE261545	GEO-spatial transcriptomics	GPL24676	1 case of early DKD	kidney tissue

Supplementary Table 2 Baseline characteristics of UK Biobank participants included in the study

	control N=779	kidney outcomes N=139	P value	Primary outcomes (renal death) N=127	P value	Secondary outcomes N=16	P value
Age (years)	60.7 (7.04)	63.5 (5.36)	<0.001	64.0 (5.02)	<0.001	59.3 (5.79)	0.236
Sex:			0.353		0.352		0.975
Female	276 (35.4%)	43 (30.9%)		39 (30.7%)		5 (31.2%)	
Male	503 (64.6%)	96 (69.1%)		88 (69.3%)		11 (68.8%)	
ethnicity:			0.558		0.462		0.154
Asian	41 (5.26%)	7 (5.04%)		7 (5.51%)		0 (0.00%)	
Black	34 (4.36%)	5 (3.60%)		3 (2.36%)		3 (18.8%)	
Mixed	1 (0.13%)	0 (0.00%)		0 (0.00%)		0 (0.00%)	
Prefer not to answer	2 (0.26%)	1 (0.72%)		1 (0.79%)		0 (0.00%)	
White	676 (86.8%)	125 (89.9%)		115 (90.6%)		13 (81.2%)	
Townsend deprivation index	-0.02 (3.56)	-0.20 (3.55)	0.593	-0.24 (3.55)	0.516	0.23 (3.84)	0.779
Education score	23.1 (19.5)	22.5 (20.2)	0.789	22.7 (20.3)	0.891	23.0 (20.8)	0.993
eGFR(mL/min/1.73 m²)	77.1 (23.2)	53.8 (21.3)	<0.001	54.1 (20.4)	<0.001	47.7 (27.3)	0.002
ACR(mg/g)	15.6 (36.1)	36.2 (74.9)	0.004	36.8 (77.8)	0.007	35.2 (42.8)	0.139
HbA1c(mmol/mol)	55.5 (18.3)	57.8 (19.3)	0.211	58.4 (19.7)	0.122	53.0 (17.6)	0.558
Diabetes duration(years)	10.9 (11.9)	17.1 (14.8)	<0.001	17.0 (15.2)	<0.001	15.3 (8.23)	0.182
Diabetic neuropathy:			0.006		<0.001		0.442
Do not know	10 (1.28%)	1 (0.72%)		1 (0.79%)		0 (0.00%)	
No	116 (14.9%)	7 (5.04%)		4 (3.15%)		3 (18.8%)	
Yes	25 (3.21%)	4 (2.88%)		3 (2.36%)		1 (6.25%)	
Diastolic blood pressure(mmHg)	83.1 (11.9)	79.0 (12.3)	0.001	78.9 (12.5)	0.001	81.8 (10.3)	0.795
Systolic blood pressure(mmHg)	147 (20.6)	149 (19.4)	0.419	149 (19.6)	0.299	149 (20.0)	0.806
BMI(kg/m²)	32.5 (6.18)	33.3 (5.93)	0.179	33.5 (6.00)	0.096	30.9 (4.46)	0.128

	control N=779	kidney outcomes N=139	P value	Primary outcomes (renal death) N=127	P value	Secondary outcomes N=16	P value
Waist circumference(cm)	106 (14.9)	109 (15.2)	0.030	110 (15.1)	0.010	102 (14.0)	0.218
Hip circumference(cm)	110 (12.6)	111 (12.5)	0.422	112 (12.8)	0.280	108 (7.36)	0.197
HDL cholesterol(mmol/L)	1.13 (0.32)	1.08 (0.31)	0.062	1.05(0.28)	0.133	1.12(0.34)	0.080
Triglycerides(mmol/L)	2.39 (1.42)	2.38 (1.47)	0.931	2.38 (1.50)	0.906	2.54 (1.49)	0.689
LDL cholesterol(mmol/L)	2.75 (0.85)	2.46 (0.82)	<0.001	2.45 (0.84)	<0.001	2.42 (0.60)	0.070
Apolipoprotein B cholesterol(mmol/L)	0.87 (0.24)	0.80 (0.22)	0.001	0.80 (0.23)	0.002	0.75 (0.16)	0.013
Lipoprotein A(mmol/L)	47.6 (51.1)	45.0 (46.8)	0.615	46.4 (47.9)	0.870	28.4 (30.2)	0.064
Glucose(mmol/L)	7.90 (3.91)	8.51 (4.35)	0.148	8.64 (4.42)	0.088	8.16 (3.76)	0.880
Hypertension:			0.179		0.495		0.057
No/Unknown	308 (39.5%)	46 (33.1%)		45 (35.4%)		2 (12.5%)	
Yes	471 (60.5%)	93 (66.9%)		82 (64.6%)		14 (87.5%)	
Hypertension duration(years)	10.7 (8.27)	13.8 (9.02)	0.002	14.2 (9.40)	0.002	10.5 (4.94)	0.614
Urea(mmol/L)	6.82 (2.64)	10.6 (5.91)	<0.001	10.5 (5.61)	<0.001	13.2 (8.45)	0.013
Albumin(g/L)	44.5 (3.17)	43.0 (3.19)	<0.001	43.0 (3.18)	<0.001	42.6 (3.41)	0.091
Cystatin C(mg/L)	1.15 (0.34)	1.67 (0.60)	<0.001	1.66 (0.58)	<0.001	1.78 (0.79)	0.012
Medications for cholesterol:			0.173		0.218		1.000
No	394 (50.6%)	61 (43.9%)		56 (44.1%)		8 (50.0%)	
Yes	385 (49.4%)	78 (56.1%)		71 (55.9%)		8 (50.0%)	
Medications for diabetes:			0.001		0.004		0.305
No	668 (85.8%)	103 (74.1%)		95 (74.8%)		12 (75.0%)	
Yes	111 (14.2%)	36 (25.9%)		32 (25.2%)		4 (25.0%)	
Medications for blood pressure:			<0.001		<0.001		0.423
No	415 (53.3%)	49 (35.3%)		45 (35.4%)		6 (37.5%)	
Yes	364 (46.7%)	90 (64.7%)		82 (64.6%)		10 (62.5%)	

	control N=779	kidney outcomes N=139	P value	Primary outcomes (renal death) N=127	P value	Secondary outcomes N=16	P value
C reactive protein(mg/L)	4.30 (5.93)	5.72 (7.89)	0.050	5.92 (8.17)	0.037	3.49 (3.39)	0.248
Alanine aminotransferase(U/L)	30.7 (21.6)	22.4 (11.8)	<0.001	22.4 (12.1)	<0.001	21.0 (9.49)	0.003
Calcium(mmol/L)	2.39 (0.12)	2.35 (0.09)	<0.001	2.35 (0.09)	<0.001	2.35 (0.13)	0.294
Potassium in urine(mmol/L)	60.6 (30.3)	52.3 (25.8)	0.001	52.7 (26.2)	0.004	49.0 (18.5)	0.040
Sodium in urine(mmol/L)	80.2 (44.6)	67.8 (37.6)	0.001	65.8 (36.2)	<0.001	80.8 (46.3)	0.831
Vitamin D(mmol/L)	39.5 (20.4)	39.9 (19.8)	0.826	40.0 (20.0)	0.805	38.2 (17.2)	0.760
Alcohol drinking status:			0.383		0.391		0.145
Current	638 (81.9%)	108 (77.7%)		99 (78.0%)		11 (68.8%)	
Never	73 (9.37%)	15 (10.8%)		14 (11.0%)		1 (6.25%)	
Prefer not to answer	1 (0.13%)	1 (0.72%)		1 (0.79%)		0 (0.00%)	
Previous	66 (8.47%)	15 (10.8%)		13 (10.2%)		4 (25.0%)	
Smoking status:			0.650		0.659		0.542
Current	84 (10.8%)	12 (8.63%)		12 (9.45%)		0 (0.00%)	
Never	317 (40.7%)	57 (41.0%)		51 (40.2%)		8 (50.0%)	
Prefer not to answer	5 (0.64%)	2 (1.44%)		2 (1.57%)		0 (0.00%)	
Previous	372 (47.8%)	68 (48.9%)		62 (48.8%)		8 (50.0%)	
Sleep hours	7.25 (1.56)	7.74 (1.71)	0.002	7.79 (1.71)	0.001	7.06 (1.57)	0.506
Enhanced PRS for type 1 diabetes	0.14 (1.15)	0.05 (1.30)	0.634	0.07 (1.23)	0.737	-0.39 (1.63)	0.461
Enhanced PRS for type 2 diabetes.	0.57 (0.97)	0.62 (0.96)	0.772	0.52 (0.92)	0.684	1.36 (0.91)	0.086

BMI: Body Mass Index; HbA1c: Glycated hemoglobin; ACR: Albumin-to-creatinine ratio; eGFR: Estimated glomerular filtration rate; HDL: High-density lipoprotein; LDL: Low-density lipoprotein; PRS: Polygenic Risk Score.

Kidney Outcomes (Composite): The overarching endpoint including both primary and secondary outcomes.Primary Outcomes (Renal Death): Specifically defined as death directly attributed to renal failure or associated kidney diseases.Secondary Outcomes: Includes clinical endpoints such as End-Stage Renal Disease (ESRD), initiation of Kidney Replacement Therapy (KRT), or a significant decline in eGFR (≥40% decrease from baseline).

Continuous variables are presented as Mean (Standard Deviation [SD]). Differences between groups were analyzed using the independent t-test or the Mann-Whitney U test, depending on the data distribution.Categorical variables are presented as Frequency (Percentage [%]). Differences between groups were evaluated using the Chi-square test or Fisher's exact test.All P-values are two-sided, with $P < 0.05$ considered statistically significant.
